# A rich catalog of C–C bonded species formed in CO_2_ reduction on a plasmonic photocatalyst

**DOI:** 10.1038/s41467-021-22868-9

**Published:** 2021-05-10

**Authors:** Dinumol Devasia, Andrew J. Wilson, Jaeyoung Heo, Varun Mohan, Prashant K. Jain

**Affiliations:** 1 Department of Chemistry, University of Illinois at Urbana-Champaign, Urbana, IL USA; 2 Department of Materials Science and Engineering, University of Illinois at Urbana-Champaign, Urbana, IL USA; 3 Materials Research Laboratory, University of Illinois at Urbana-Champaign, Urbana, IL USA; 4 Department of Physics, University of Illinois at Urbana-Champaign, Urbana, IL USA; 5 Beckman Institute for Advanced Science and Technology, University of Illinois at Urbana-Champaign, Urbana, IL USA; 6 Present Address: Department of Chemistry, University of Louisville, Louisville, KY USA

**Keywords:** Photocatalysis, Raman spectroscopy, Nanophotonics and plasmonics, Nanoparticles

## Abstract

The understanding and rational design of heterogeneous catalysts for complex reactions, such as CO_2_ reduction, requires knowledge of elementary steps and chemical species prevalent on the catalyst surface under operating conditions. Using in situ nanoscale surface-enhanced Raman scattering, we probe the surface of a Ag nanoparticle during plasmon-excitation-driven CO_2_ reduction in water. Enabled by the high spatiotemporal resolution and surface sensitivity of our method, we detect a rich array of C_1_–C_4_ species formed on the photocatalytically active surface. The abundance of multi-carbon compounds, such as butanol, suggests the favorability of kinetically challenging C–C coupling on the photoexcited Ag surface. Another advance of this work is the use of isotope labeling in nanoscale probing, which allows confirmation that detected species are the intermediates and products of the catalytic reaction rather than spurious contaminants. The surface chemical knowledge made accessible by our approach will inform the modeling and engineering of catalysts.

## Abstract (web summary)

In situ vibrational spectroscopy affords a powerful tool for probing elementary chemical processes on catalytic surfaces. Using surface enhanced Raman scattering, authors identify an array of multicarbon species formed on a Ag nanoparticle catalyst in plasmon-driven reduction of CO_2_ in water.

## Introduction

Traditionally, heterogeneous catalysts are tested, screened, and optimized on the basis of the products of the catalytic reaction that are formed in the bulk phase. Yet, it is the adsorbates, intermediates, and species formed on the surface of the catalyst that are the fundamental signifiers of how a catalyst functions. Quantum-chemical modeling of a catalyst relies on information of surface species, comprehensive knowledge of which under operando conditions is often poor. As a result, despite the success of empirical catalyst discovery, mechanistic understanding has remained limited. This is especially the case for catalytic chemistry such as the CO_2_ reduction reaction (CO_2_RR), where multiple steps and alternate pathways are involved. Chemical interrogation of the surface in situ under catalytic turnover conditions is important for advancing molecular-level understanding and enabling knowledge-driven modeling of catalysts^1^. Here, we characterize in comprehensive detail the surface species formed on a CO_2_RR photocatalyst under operating conditions. In addition to the use of realistic aqueous conditions for this reaction, our methodology combines nanoscale spatial resolution^2–8^ and sub-second temporal resolution, which allows us to capture ephemeral intermediates that are otherwise hidden in measurements averaged over larger ensembles^9–15^. The vast array of species captured in spectra is identified using an automated procedure that queries a database of carbonaceous compounds we developed for this study. A fuller picture of the chemical activity on the photocatalyst surface is thus obtained. Another important feature of our study is the development of isotopological validation of the nanoscale surface interrogation, which ensures that detected species are indeed the intermediates and products of the catalytic reaction rather than artefacts and contaminants that commonly plague surface interrogations in realistic reaction media.

We apply our approach to a plasmonic nanoparticle (NP)-based photocatalyst for the CO_2_RR. The solar light-driven conversion of CO_2_ and water into energy-dense, value-added multi-carbon molecules is of considerable interest as a technology for renewable energy generation and chemical manufacturing^16^. In particular, the fixation of CO_2_ into liquid hydrocarbons and alcohols is desirable due to the high energy density and easy transportability of these products^17,18^. Consequently, there is an ongoing quest for a synthetic photocatalyst that facilitates the reduction of CO_2_^19–21^ and the coupling of the reduced intermediates into multi-carbon molecules^22–24^. Such C–C coupling, in particular, is kinetically unfavorable in most cases.

Plasmonic NPs of Au, Ag, and Cu have been found to photocatalyze CO_2_RR due to the action of energetic electron–hole pairs formed by the decay of localized surface plasmon resonances (LSPRs)^25–29^ excited in the NPs^30–38^. Further, Ag NPs serve as resonant optical antennas that concentrate electromagnetic fields at their surfaces^39^ and lead to intense surface-enhanced Raman scattering (SERS) from molecules present at the surface of the NP^40^. SERS is a powerful tool for selectively probing the chemistry occurring at the surface of a plasmonic NP^41–49^. Previous work from our group and others has pushed the limits of SERS and used it for in situ high spatial resolution probing of chemical reactions without requiring the use of fluorogenic probes and reactions^34,50–56^. For the current study, we expanded single-NP-level SERS to a liquid water reaction medium and monitored plasmon-excitation-driven CO_2_RR events on Ag NPs under these conditions. We found an unexpectedly wide range of C_1_–C_4_ species, including hydrocarbons, alcohols, and oxygenates, to be formed on the NP surface under light excitation. The observed prevalence of C–C coupling on the Ag surface is striking and calls for further exploration and optimization of light-driven CO_2_RR on Ag NPs. This work is also an experimental advance in chemical imaging in a label-free, high spatiotemporal resolution, and reliable manner. Our approach can be extended to the study of other light-driven reactions on surfaces in fluid media.

## Results

### Single-NP-level SERS probing in an aqueous medium

**Fig. 1:**
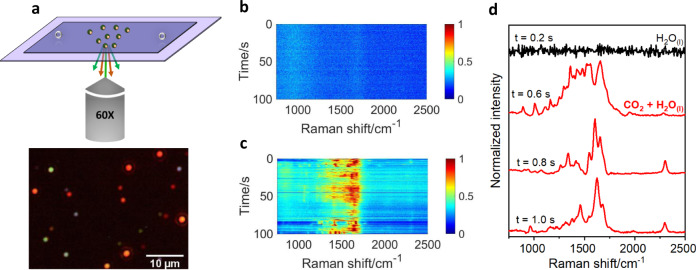
In situ SERS probing of plasmon-excitation-driven CO_2_RR in an aqueous medium. **a** Top panel shows a schematic of the single-NP-level SERS spectroscopy set-up used for probing CO_2_ photochemistry on a Ag NP in water, i.e., H_2_O_(1)_. The Ag NP was immobilized inside a microfluidic cell placed in an inverted optical microscope and subject to continuous-wave focused laser excitation. Raman scattering was continuously collected using a ×60 objective. The bottom panel shows a representative dark-field scattering image of the substrate coated with spatially resolvable Ag NP emitters. Representative spectrograms showing time series of in situ SERS spectra acquired from an individual Ag NP scatterer in **b** CO_2_-untreated H_2_O_(1)_ and **c** CO_2_-saturated H_2_O_(1)_. The SERS intensity is coded by the color, as indicated by the legend. **d** Spectral slice, corresponding to *t* = 0.2 s, from the plot in **b** shows the lack of any vibrational modes. Spectral slices, corresponding to *t* = 0.6, 0.8, and 1.0 s from the plot in **c** show the spectral dynamics taking place in the presence of CO_2_-saturated H_2_O_(1)_ under LSPR excitation. The spectra were normalized to a [0, 1] range prior to plotting in the form of spectrograms in **b**, **c**. The spectra were baseline-subtracted and normalized to a [0, 1] range prior to plotting in a vertically stacked manner in **d**. The baseline was determined by polynomial fitting. Source data are provided as a source data file.

The CO_2_RR photocatalyst and SERS platform consisted of Ag NPs synthesized by the Lee–Meisel method^57^. The as-synthesized colloid contains NPs of a variety of sizes and shapes and surface-coated with citrate ligands (Supplementary Figs. 1 and 2). The colloidal NPs were drop casted onto a clean coverslip (Supplementary Fig. 3). We ensured that the area density was low enough for discrete scatterers to be spatially resolvable in diffraction-limited optical microscopy using a ×60 objective (Fig. 1a and Supplementary Figs. 4 and 5). The discrete scatterers are typically single Ag NPs, dimers, or trimers (Supplementary Figs. 4–6) as determined by scanning electron microscopy (SEM) and transmission electron microscopy (TEM). The NP-coated substrate was then subjected to ultraviolet (UV) radiation to photo-oxidize and clean organic ligands and contaminants off the NP surface (Supplementary Fig. 8a). This cleaning procedure ensures that SERS spectroscopy probes only CO_2_ photoreduction activity and is not complicated by the photochemical reactions of citrate or other carbonaceous contaminants on the Ag NP surface^54^. UV light treatment has the potential to oxidize the Ag; extensive oxidation of the Ag NP preclude catalysis and SERS. Therefore, substrate-bound NPs subjected to photo-oxidative cleaning were examined by TEM, which shows that the NPs are comprised of metallic Ag and do not have a Ag oxide shell on the surface (Supplementary Fig. 6). The morphology of the NPs does not appear to have been modified by UV light illumination. However, a Ag sub-oxide is indicated to be present by high-sensitivity surface characterization by SERS (Supplementary Fig. 8b). Following photo-oxidative cleaning (Fig. 1b and Supplementary Figs. 8 and 9), the NP-coated coverslip was integrated into a home-built microfluidic cell (Supplementary Fig. 3). For SERS spectroscopy, the microfluidic cell was filled with deionized (DI) water saturated with CO_2_ and mounted on an inverted microscope (Fig. 1a). Despite the presence of the liquid layer covering the Ag NPs, we were able to selectively probe chemical activity at the surface of the Ag NPs. The sharp decay of the electric field enhancement of Raman scattering over a distance on the order of 1–10 nm from the NP surface^58,59^ ensures that the measured SERS signals emanate specifically from surface species; there is little to no contribution of SERS from the tens-of-μm-thick liquid layer.

Similar to our previous study^34,55^, we identified by dark-field scattering imaging individual diffraction-limited Ag NP scatterers that were spatially resolvable from other NP scatterers (Fig. 1). One such scatterer was then subjected to a beam of a continuous-wave (CW) 514.5 nm laser focused using a ×60 objective. Note that a typical scatterer chosen for SERS may not be a single nanosphere; in fact, it is more likely an anisotropic NP, a NP dimer, or a NP trimer (Supplementary Figs. 4–7). Such structures dominate dark-field scattering, display overlap of LSPR bands with the 514.5 nm laser excitation (Supplementary Fig. 7)^34^, and also exhibit large SERS signals due to the electric field hotspots supported at their inter-NP junctions^34,60^ or sharp tips. Excitation of LSPRs by the 514.5 nm laser initiated photochemistry of CO_2_ on the Ag NP surface. The 514.5 nm laser was also the Raman excitation source. SERS spectra arising from species formed on the Ag NP surface in the course of CO_2_ photochemistry were acquired continuously under LSPR excitation with an acquisition time of 200 ms per frame. An interrogated scatterer does not migrate during the course of the acquisition, as we confirm at the end of each experiment. Using this method, 96 individual Ag NP scatterers were interrogated. More details can be found in the “Methods” section and Supplementary Information document. Examples of SERS movies acquired by the method described are provided as Supplementary Movies 1 and 2.

### Spectral tracking of plasmon-excitation-driven CO_2_RR events

The continuously acquired SERS spectra capture snapshots of discrete photochemical events occurring on the Ag NP surface under continuous LSPR excitation. This is best depicted by time series of SERS spectra plotted as a spectrogram (Fig. 1c, d and Supplementary Fig. 10a, b). SERS spectra acquired from individual plasmon-excited Ag NPs in CO_2_-saturated water show dynamics in the 750–1800 cm^−1^ range where vibrational signatures of hydrocarbons, alcohols, acids, and esters are known to appear. In addition to fluctuations in the intensities of vibrational bands (Supplementary Fig. 10c), specific vibrational modes appear and disappear signifying the formation of transient adsorbates and intermediates in photodriven CO_2_RR at the NP surface. SERS spectra of individual plasmon-excited Ag NPs in water that is not intentionally saturated with CO_2_ showed no vibrational bands in the fingerprint region (Fig. 1b, d and Supplementary Fig. 9). The result of this control experiment rules out the possibility of contaminants in water or adventitious adsorbates and ligands on the Ag NP surface causing the dynamic SERS activity observed in CO_2_-saturated water.

### Uncovering the array of surface species formed

From 96 individual Ag NP scatterers under photodriven CO_2_RR conditions, we collected 98,000 SERS spectra, providing us with a comprehensive profile of the surface adsorbates and intermediates formed in the reaction. An examination of the spectra indicated that the range of species captured in the spectra is vast and includes many that are not known from bulk catalytic studies to be produced by Ag-catalyzed CO_2_RR. To allow identification of these species, we used a database-query approach. From an extensive search of the CO_2_RR literature^61–74^, we created a database (Supplementary Table 1 and vibrational assignments in Supplementary Tables 2–24) consisting of organic molecules that putatively can be generated by reactions starting from CO_2_. The list includes both species formed by direct transformations of CO_2_ and those formed indirectly by reactions between species formed directly from CO_2_. This database can be further extended by us or others by inclusion of other probable products. Raman vibrational modes of all species in the database were calculated by density functional theory (DFT). An automated peak-matching algorithm was employed to assign each acquired spectrum to a species from this reference database (see Section II in Supplementary Information). In a subset of cases, an acquired spectrum could not be assigned solely to one species; such a spectrum likely captured more than one species simultaneously present on the Ag surface during the 200 ms acquisition time.

**Fig. 2:**
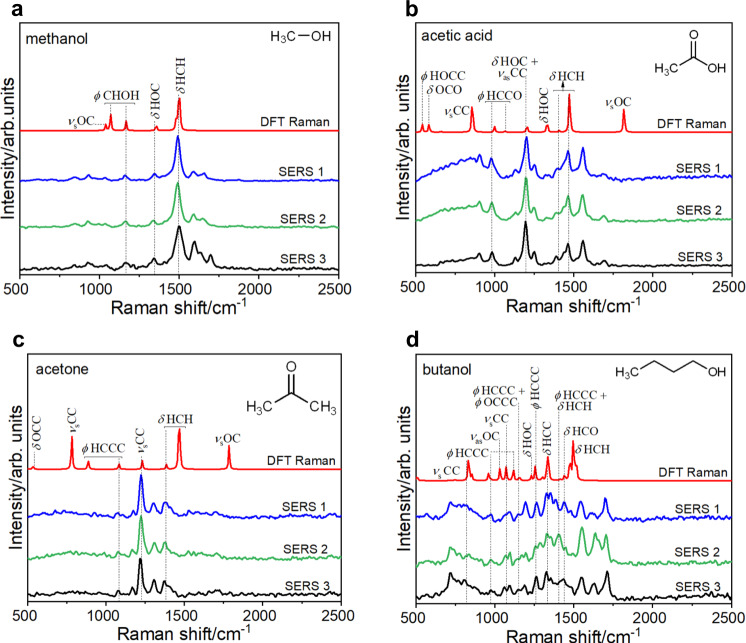
Species detected on Ag NPs in the course of plasmon-excitation-driven CO_2_RR in water. Three representative examples of measured SERS spectra (blue, green, and black traces) that correspond to the detection of **a** methanol, **b** acetic acid, **c** acetone, and **d** butanol. In each case, the DFT-calculated Raman vibrational spectrum, which served as the basis of the assignment of the measured SERS spectra, is also shown by the red trace. The major vibrational modes are labeled using the following symbols: *ϕ*—torsion; *ν*_s_—symmetric stretching; *ν*_as_—asymmetric stretching; *δ*—bending. Spectra are shown vertically stacked for clarity. SERS spectra were baseline-subtracted, subjected to smoothing by a Savitzky–Golay filter with a window of 5 points and a polynomial of order 3, and normalized to a [0, 1] scale prior to plotting in a vertically stacked manner. The baseline was determined by polynomial fitting. DFT-computed Raman spectra were normalized to a [0, 1] scale, and the *y*-axis quantity was magnified (by a scale factor of 4 in **a**, **b** and a factor of 10 in **c**, **d**) prior to plotting. Source data are provided as a source data file.

It is known that electrochemical CO_2_RR on Ag predominantly yields CO^75–78^. So, we scanned the large collection of spectra for the presence of CO, the stretching vibrational mode of which is expected to appear in the 1800–2200 cm^−1^ region, with the exact location dependent on its adsorption motif. But a small fraction of spectra (~0.1%) obtained from the 96 individual NP scatterers corresponded to the detection of CO. Our analysis showed an abundance of other organic compounds, including multi-carbon ones. Thus, the surface species distribution uncovered here is richer than the typical bulk product distribution observed for electrochemical CO_2_RR on Ag^75–77^. The detected species were grouped on the basis of the number of C atoms constituting them. The distribution was dominated by C_2_–C_4_ compounds, indicative of the prevalence of C–C coupling events on the NP surface under plasmon-excitation conditions. Representative examples of in situ SERS spectra that captured various C_1_–C_4_ species are shown in Fig. 2 and Supplementary Figs. 11–19.

The CO_2_RR requires a counter oxidation reaction. Under the conditions of our in situ SERS experiments, possible counter reactions are the oxidation of water to oxygen (O_2_) or hydrogen peroxide (H_2_O_2_), Ag oxidation^34^, and Ag hydroxylation. We detected in SERS spectra instances of detection of O_2_ (Supplementary Fig. 19c) and H_2_O_2_ (Supplementary Fig. 19b), which are indicative of water oxidation.

### Isotopological validation of CO_2_RR origin of surface species

Surfaces in realistic fluid media can often be contaminated with hydrocarbons and other organic compounds from the environment. Ligands leftover from NP synthesis can be another spurious source of species detected on the Ag NP surface. It was therefore important to ensure that the observed surface species were not artefactual and that the precursor of the detected species was indeed CO_2_. We used isotope labeling^79,80^ for this verification. We conducted SERS probing of plasmon-excitation-driven CO_2_RR on individual Ag NP scatterers in an aqueous medium saturated with ^13^C-labeled CO_2_. Species generated from ^13^CO_2_ are expected to have vibrational spectra with their mass-sensitive modes shifted in frequency with respect to the ^12^C isotopologs. For each species, the expected isotopological frequency shift, *ν*(^13^C) − *ν*(^12^C), was calculated by DFT for every vibrational mode in the Raman spectrum. We determined from experimental SERS spectra isotopological shifts for all detected species (see Supplementary Information) and compared them with corresponding DFT-predicted values, as shown in Supplementary Tables 25–45. For the surface species detected, the isotopological shifts observed in experiments have the same sign and magnitude, within one standard deviation (SD), as the corresponding DFT-predicted values for most of the vibrational peaks.

**Fig. 3:**
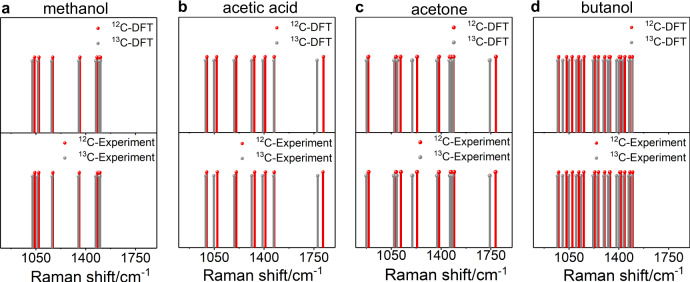
Isotopological validation of CO_2_RR origin of surface species. Spectral barcodes for the DFT-computed Raman spectra (top row) and experimental SERS spectra (bottom row) for ^12^C (red lines) and ^13^C (gray lines) isotopologs of **a** methanol, **b** acetic acid, **c** acetone, and **d** butanol. Each vertical line in a barcode indicates the peak wavenumber of a vibrational mode. The number of SERS spectra that led to the experimental barcodes are **a** 119 and 43, **b** 106 and 59, **c** 243 and 177, and **d** 1062 and 836 for the ^12^C and ^13^C isotopologs, respectively. Source data are provided as a source data file.

For clear visualization of the isotopological validation, measured SERS spectra of ^12^C and ^13^C isotopologs of each species are plotted in the form of spectral barcodes. An experimental barcode consists of vertical lines, which denote the averaged peak wavenumber for each SERS band in the spectrum. The method for barcode construction is detailed in Supplementary Information (Supplementary Fig. 20). A reference barcode was also constructed from the DFT-computed Raman spectrum for each species. For all the major surface species (Fig. 3 and Supplementary Figs. 21–25), the pattern of isotopological shifts is in close agreement between the experimental and reference (DFT-computed) barcodes. Thus, we validate our assignments of spectra to molecular species and also corroborate that CO_2_ is the precursor of the detected surface species.

## Discussion

**Fig. 4:**
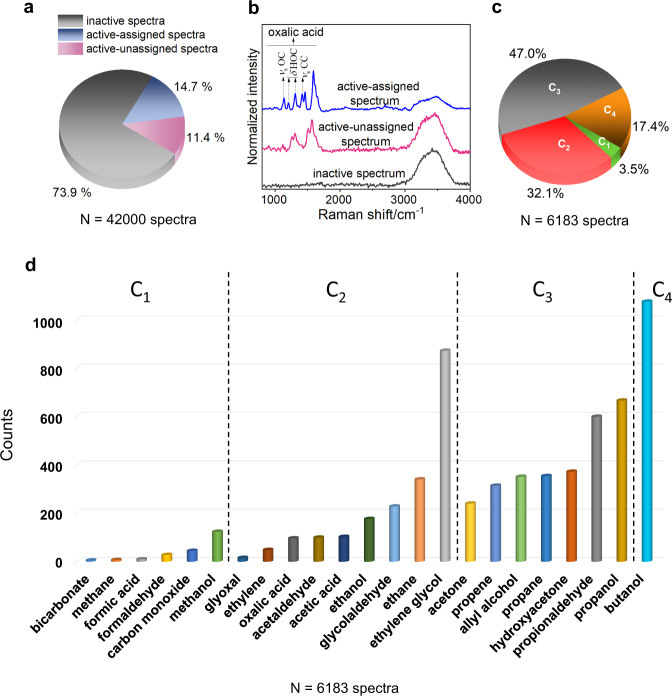
Profile of surface species observed in CO_2_RR by in situ nanoscale SERS. **a** Pie chart showing the percentage distribution of inactive SERS spectra, active spectra that were assigned to a species in the database, and active spectra that were unassignable. This distribution was obtained by an analysis of 42,000 in situ SERS spectra collected from 40 individual Ag NP scatterers in ^12^CO_2_-saturated H_2_O_(1)_ under plasmonic excitation. **b** Representative examples of SERS spectra in each of these three classes, plotted in a vertically stacked manner. The broad band at ca. 3400 cm^−1^ in all three spectra is due to Raman scattering from H_2_O_(1)_ in the medium. The active, assigned SERS spectrum shown as an example captured oxalic acid, the major vibrational modes of which are labeled by the following symbols: *ν*_s_—symmetric stretching; *δ*—bending. SERS spectra were baseline-subtracted, subjected to smoothing by a Savitzky–Golay filter with a window of 5 points and a polynomial of order 3, and normalized to a [0, 1] scale prior to plotting in a vertically stacked manner. The baseline was determined by polynomial fitting. **c** Pie chart showing the percentages of active, assigned spectra that captured a C_1_, C_2_, C_3_, and C_4_ species. **d** Bar plot showing for each surface species the number of detection events across the set of active, assigned spectra. Each species is shown by a bar of a different color. The species are grouped into C_1_, C_2_, C_3_, and C_4_ categories; within each category, they are listed in the order of their prevalence. *N* in **a**, **c**, and **d** represents the total number of spectra that led to the plot. Source data are provided as a source data file.

With the in situ nanoscale SERS probing method fully validated, we discuss the major findings about chemical species formed in the plasmon-excitation-driven CO_2_RR on Ag. Of the 42,000 spectra collected from 40 individual Ag NP scatterers in ^12^CO_2_-saturated water, 74% of the spectra showed no SERS peaks (Fig. 4a, b). About 15% of the spectra showed activity (Fig. 4a, b) and were assignable to a species in the reference database (Fig. 4d). The remaining 11% were non-assignable (Fig. 4a, b) as per our mode-match algorithm; it is possible that some of these spectra captured a species not present in the database or they captured species that have <3 modes in the fingerprint region.

A similar distribution was seen for the 56,000 spectra acquired from 56 individual Ag NP scatterers in ^13^CO_2_-saturated water (Supplementary Fig. 26). The distribution of surface species by detection counts in the active, assigned spectra is shown in Fig. 4d. It must be noted that the relative counts of species are not a quantitative representation of their relative abundance because the likelihood of detection of a species is influenced by factors such as its Raman cross-section, chemical enhancement factor, orientation and location with respect to the electromagnetic field hotspot, and surface residence time. Furthermore, the identification of a species may be positively biased by the number of Raman vibrational modes in its SERS spectrum. Because of the conditions imposed by our algorithm, longer hydrocarbons and alcohols, which have a larger number of modes, may be more likely to be identified than smaller molecules like methane, which have fewer modes.

In situ nanoscale SERS (Fig. 4d) shows the surface of the photocatalyst to be abundant in multi-carbon hydrocarbons and alcohols, which are kinetically challenging to produce in CO_2_RR. In electrocatalytic CO_2_RR on Ag^81,82^, H_2_—formed by competing HER—and CO^83,84^ are the most common bulk products on Ag; there are some reports of the observation of formate^76,84^, methane^85–87^, ethylene^87^, methanol, and ethanol^77^, albeit in smaller yields. In our study, in addition to these previously reported products, we detected on the surface C_2_ compounds such as ethylene glycol, glyoxal, glycolaldehyde, and oxalic acid; C_3_ compounds such as propane, propanol, and acetone; and the C_4_ compound, butanol. There are no previous reports of the formation of C_3_ or C_4_ products in Ag-catalyzed CO_2_RR. These higher hydrocarbons and alcohols have been reported on other metal catalysts and under special conditions such as high-pressure electrochemistry^69,88–112^. Another species of note detected in our study is oxalic acid (Supplementary Fig. 12c), which is known to be formed in photosynthesis. The dominance of multi-carbon species in the distribution (Fig. 4c, d and Supplementary Fig. 26) leads us to infer that C–C coupling is favorable on the plasmon-excited NP surface.

The formation of the wide variety of C_2+_ chemical species on a Ag NP surface in the presence of visible light excitation and water, without an applied potential, is striking. The atypical profile of multiplex species found here may be an outcome of one or more unique features of our methodology. First, the higher surface sensitivity of SERS combined with the 200 ms temporal resolution and nanoscale sampling volume employed here is quite unlike the bulk-level characterization methods used previously. Second, the catalytic activation method utilized in this study is considerably different from the typical conditions in photocatalytic or electrocatalytic CO_2_RR. Under the focused plasmonic excitation with an intensity of ~10^8^ W m^−2^ we use, the average time between absorbed photons is ~700 fs (Supplementary Table 46), which is shorter than the 1-ps-scale time constant^113^ of electron–hole recombination. These conditions can favor multiphoton excitation and re-excitation of electron–hole pairs (Supplementary Fig. 27), resulting in the generation of highly energetic carriers (Supplementary Fig. 27) akin to those generated by UV light. Such effects may be responsible for the richer photochemistry observed here. While a product profile that is abundant in high-value hydrocarbons and alcohols is desirable, it must be acknowledged that what is measured in these experiments are trace amounts of species generated on the surface that do not necessarily survive or culminate into the bulk product profile. However, there is a possibility that, by appropriate engineering of the Ag NP catalyst and the reaction conditions, it may be possible to scale-up the formation of these promising multi-carbon species and recover them as products in the bulk phase.

These findings highlight the potential of in situ nanoscale SERS probing for providing insights into elementary reaction steps and species prevalent on a photocatalyst surface under operando conditions. This insight, which is typically inaccessible by conventional bulk-level interrogation of heterogeneous catalysts, is made possible by the high spatiotemporal resolution, surface sensitivity, and reliability of our approach. Noble metals that are effective SERS substrates are naturally suited for highly sensitive interrogation by SERS; however, the method has potential to be extended^114^ to other classes of catalysts and photocatalysts. A finding of general importance made here is that the profile of surface species formed under catalytic conditions can differ dramatically from the expected profile of products the catalyst is known to yield. This knowledge will guide more accurate computational modeling, advance mechanistic understanding of the catalytic process, and revolutionize the way catalysts are designed, especially for multi-step, multi-pathway reactions such as CO_2_RR.

## Methods

### Sample preparation for SERS experiments

#### Synthesis of Ag NPs

Ag NPs for the SERS studies were synthesized by the Lee–Meisel method^57^. Briefly, 45 mg of AgNO_3_ was added to 250 mL of boiling DI water. A solution of trisodium citrate was prepared by dissolving 50 mg of the compound in 5 mL of DI water under stirring. After 5 min of stirring, the entire 5 mL of the citrate solution was added to the AgNO_3_ solution under stirring. Boiling of the mixture was continued for 1 h. After the addition of the citrate solution, the mixture started changing its color from bright yellow to greenish yellow. This Ag colloid was then allowed to cool to room temperature and stored in fridge. The measured extinction spectrum of the as-synthesized Ag NP colloid is shown in Supplementary Fig. 1.

#### Preparation of microfluidic reaction cells

SERS-based tracking of plasmon-excitation-driven CO_2_RR events was performed using a microfluidic reaction cell. To construct a microfluidic cell (Supplementary Fig. 3), holes of 1 mm diameter were drilled in a glass coverslip (24 × 60 mm^2^, VWR SuperSlips, no. 1). The coverslip was cleaned by sonication in DI water for 10 min and then dried in air. Then 10 µL of as-synthesized Ag NPs, diluted by the addition of 1 mL of DI water, was drop casted onto the coverslip, which was then heated on a hot plate for drying. This procedure has been optimized to obtain well-isolated Ag NP scatterers suitable for our single-NP-level SERS studies. After multiple rinses with DI water and drying under N_2_, this NP-coated coverslip was exposed to UV light from a Hg lamp for ca. 10 min to remove any organic contaminants and ligands by UV photo-oxidation. This cleaning procedure ensures that Ag NPs are free of ligands, which would otherwise complicate SERS spectra, and have accessible active surfaces for catalysis. Representative examples of a SERS spectrum from an “unclean” NP scatterer (with surface-adsorbed citrate ligands) and a “clean” NP scatterer (with no surface-adsorbed citrate ligands or other contaminants) are shown in Supplementary Fig. 8a. The length of the UV light treatment was optimized by multiple trials so that the NPs exhibit ligand-free SERS spectra while ensuring that the Ag NP surfaces do not get photo-oxidized, which would mar their catalytic activity. After UV light exposure, the NP-coated coverslip was rinsed with DI water and dried in air. Then the coverslip was placed onto another clean coverslip (24 × 60 mm^2^, VWR SuperSlips, no. 1 with no drilled holes) spaced by double-sided tape, which has a thickness of 10s of μm, and glued using an epoxy glue to form the microfluidic cell.

#### Assessment of cleanliness of Ag NPs

For reliable SERS study of plasmon-excitation-driven CO_2_RR on Ag NPs, the NPs must have clean surfaces. Although we employed UV photo-oxidation to clean the NP surfaces, it was important to assess the resulting cleanliness. For such an assessment, a separate set of studies was performed where SERS spectra were acquired from individual Ag NP scatterers in CO_2_-untreated DI water. The microfluidic reaction cell was filled with DI water by using a syringe to inject DI water through the drilled holes. The cell was then mounted on the stage of a Olympus IX51 inverted microscope. Spatially isolated scatterers were identified by dark-field scattering imaging. Once an individual scatterer was identified, it was subjected to a 9–10 mW beam of a CW 514.5 nm laser focused using a ×60 Olympus UPlanApo water immersion objective. At the same time as the laser excitation was turned on, the continuous acquisition of SERS spectra from the scatterer was started. SERS spectra were collected using the same objective and filtered by a ca. 530 nm long-pass emission filter before being dispersed by a Princeton Instruments Acton SP2300 spectrometer equipped with a 300 g mm^−1^ grating. The dispersed light was then detected in a spectrally resolved manner by a Princeton Instruments Pylon 100B liquid nitrogen-cooled charged coupled device (CCD). Using the software Winspec, a 500-frame-long movie of SERS spectra (scattered intensity vs. wavelength) was collected with an acquisition time of 200 ms per frame. In Winspec, the wavelength of the scattered light, in nm units, was converted into a Raman shift in wavenumber (cm^−1^) units. In this manner, 105 individual Ag NP scatterers were interrogated. An example movie of SERS spectra acquired from an individual Ag NP scatterer in CO_2_-untreated DI water is provided (Supplementary Movie 1). These spectral movies from all the scatterers interrogated were analyzed to determine the presence of surface ligands and/or contaminants on the NP scatterers. The statistics from this analysis are shown in Supplementary Information. To serve as examples, for a few cases, the time series of continuously acquired SERS spectra from an individual scatterer is plotted as a spectrogram (Fig. 1b and Supplementary Fig. 9), which is a color-coded map of the intensity plotted as a function of Raman shift and time on the *x* and *y* axes, respectively. Prior to plotting, the SERS spectrum at each time point was normalized to a [0, 1] scale.

#### SERS studies of CO_2_RR activity of Ag NPs

For in situ SERS studies of plasmon-excitation-driven CO_2_RR on Ag NPs, the following procedure was employed. The microfluidic cell bearing the cleaned NPs immobilized on a glass coverslip was filled with DI water, which had been saturated with ^12^CO_2_ or ^13^CO_2_ by bubbling ^12^CO_2_ (99.999% purity) or ^13^CO_2_ (<3 atom% ^18^O, 99.0 atom% ^13^C) gas, respectively, through it for 20 min at a flow rate of 15–20 cc min^−1^. The reaction medium prepared in this manner has a bulk pH of ~4.4.

The liquid-filled cell was mounted onto the stage of an Olympus IX51 inverted microscope. First, a spatially isolated Ag NP scatterer was identified by dark-field scattering imaging. A 9–10 mW beam of a 514.5 nm laser line was focused onto the identified scatterer using a ×60 Olympus UPlanApo water immersion objective. At the same time as the laser excitation was turned on, the continuous acquisition of SERS spectra from the scatterer was started. Spectra were collected using the same objective and filtered by a ca. 530 nm long-pass emission filter before being dispersed by a Princeton Instruments Acton SP2300 spectrometer equipped with a 300 g mm^−1^ grating. The dispersed light was then detected in a spectrally resolved manner by a Princeton Instruments Pylon 100B liquid nitrogen-cooled CCD. Using the software Winspec, 1000-frame-long movies of SERS spectra (scattered intensity vs. wavelength) were collected with an acquisition time of 200 ms per frame. In Winspec, the wavelength of the scattered light, in nm units, was converted into a Raman shift in wavenumber (cm^−1^) units. In a typical SERS experiment performed in ^12^CO_2_- or ^13^CO_2_-saturated water, acquired spectra showed considerable dynamics in the fingerprint region (500–1800 cm^−1^): as a function of time, vibrational bands in the spectra fluctuated in intensity and certain vibrational bands appeared and disappeared. This dynamics is indicative of the formation of transient species and intermediates, i.e., photodriven CO_2_RR activity, on the NP surface.

### Data analysis

#### DFT-computed Raman spectra

Raman spectra of all the species in the database were calculated by DFT implemented on the software, Gaussian 09^115^. The molecular structure of the species was constructed in Gaussian and then its geometry was optimized by DFT using the B3LYP functional and the 6-311G++ (d, p) basis set. The Raman spectrum (harmonic frequencies) of the optimized structure was calculated using the same functional and basis set. All the calculations were performed for structures that correspond to gas phase species at a temperature of 298.150 K and a pressure of 1 atm. Calculations were performed for the normal (^12^C) and the heavy (^13^C) isotopologs of each species. DFT-computed frequencies of Raman vibration modes are listed in Supplementary Tables 2–24 species by species. Mode frequencies for the ^12^C isotopolog are listed in the leftmost column and those for the ^13^C isotopolog are listed in the rightmost column of each table. These tabulated frequencies were used for the assignment of experimental SERS spectra to species.

#### SERS peak analysis

To determine peaks in acquired SERS spectra associated with vibrational modes, we subjected the spectra to derivative analysis in MATLAB. Each raw spectrum (intensity as a function of the Raman shift) was first smoothed in the software MATLAB using a Savitzky–Golay filter with a window size of 5 and the order of the polynomial set to 1. Then the first derivative of the intensity was calculated. The derivative spectrum was also smoothed using a Savitzky–Golay filter with window size of 5 and an order of the polynomial of 1. The wavenumber locations of peaks in the acquired spectrum were determined by identifying all zero-crossing points in the smoothed derivative spectrum that match two criteria. First, the zero-crossing point ought to lie on a negative-slope region of the derivative spectrum. Second, this negative-slope region ought to begin at an intensity that is above a threshold set to 3× the standard deviation of the scattering intensity of the smoothed derivative spectrum calculated between 4000 cm^−1^ and 7000 cm^−1^, where no vibrational modes are observed and only noise contributes to the spectrum.

#### Spectral assignment

Once the wavenumber locations of all peaks in a SERS spectrum were determined, these wavenumbers were compared with the DFT-calculated Raman frequencies of vibrational modes of all species in our database. A peak is considered to correspond to a vibrational mode if the wavenumber location of the peak lies within a tolerance range of ±10 cm^−1^ around the DFT-computed Raman frequency of the mode. A spectrum is assigned to a species if three or more peaks in the SERS spectrum correspond to the Raman vibrational modes of that species. This method was followed to analyze and assign over 98,000 spectra acquired from the 96 NP single-NP-level scatterers interrogated across multiple experiments. Note that relative intensities of vibrational peaks in a DFT-computed Raman spectrum do not model the relative intensities of vibrational peaks in a corresponding SERS spectrum; hence, only the peak locations are used for assigning a measured spectrum to a species.

The reference database consists of simple C_1_–C_4_ hydrocarbons, alcohols, and oxygenates. Naturally these species are similar in terms of the functional groups and bonding motifs they contain. So, there is potential for spectral overlap between vibrational features of different species, especially given the considerable size of the database. It was necessary to ensure that such spectral overlap does not affect the reliability of our spectral assignments. The algorithm we used considers the appearance of a peak in the experimental SERS spectrum within a ±10 cm^−1^ range around the DFT-computed frequency of a Raman mode to be a mode-match. For a spectrum to be assigned to a particular species, a minimum of three such matches between the experimental SERS spectrum and the DFT-computed Raman spectrum is required. Note that we did not require a match between all modes of a species, because not all DFT-computed Raman vibration modes necessarily appear in an experimental spectrum corresponding to a species. The three-mode-match threshold we use reduces the effect of potential overlap between modes of different species and increases the likelihood of a unique assignment of a spectrum. In a subset of the cases, a spectrum gets assigned to more than one species, in which case all these species are deemed to be detected. Manual assignment of a set of 500 experimental SERS spectra showed that automated spectral assignment based on the aforementioned criteria is reliable. It must be noted that the three-mode-match assignment method is inappropriate for finding spectra which capture CO because CO has only one vibrational mode. Therefore, for identifying spectra containing the CO mode, we ran a separate analysis for the full set of spectra obtained with ^12^CO_2_-saturated water using the method described above but with a single mode-match criterion and an increased tolerance of ±100 cm^−1^.

## Supplementary information

Supplementary Information

Supplementary Movie 1

Supplementary Movie 2

Description of Additional Supplementary Files

## Data Availability

Underlying and supporting data are also provided in the form of tables and figures in the Supplementary Information document. Additional information is available from the authors upon reasonable request. Source data are provided with this paper.

## Source data

Source Data

## Code availability

All codes and algorithms are available from the authors upon reasonable request.
