# Supplementary material for: A rich catalog of C–C bonded species formed in CO2 reduction on a plasmonic photocatalyst
Source: Nat Commun. 2021 May 10;12:2612. doi: 10.1038/s41467-021-22868-9 (PMC8110802; doi:10.1038/s41467-021-22868-9)
Supplement: Supplementary file 1 — Supplementary Information [file 41467_2021_22868_MOESM1_ESM.pdf]

SUPPLEMENTARY INFORMATION

A rich catalog of C–C bonded species formed in CO_2 reduction on a plasmonic photocatalyst

Dinumol Devasia[†], *Andrew J. Wilson*[†,~], *Jaeyoung Heo*[‖], *Varun Mohan,*[‖]

and Prashant K. Jain[†,‡,#,$,*]

[†]Department of Chemistry, [‖]Department of Materials Science and Engineering, [‡]Materials Research Laboratory, [#]Department of Physics, and [$]Beckman Institute for Advanced Science and Technology, University of Illinois at Urbana-Champaign, Urbana, Illinois 61801, United States

~Present Address: Department of Chemistry, University of Louisville, Louisville, Kentucky, 40292, United States

*Corresponding author E-mail: jain@illinois.edu

Table of Contents

I. Sample preparation for surface-enhanced Raman scattering (SERS) experiments

II. Reference database and modes for the assignment of SERS spectra to species

III. SERS spectra of surface species detected

IV. Isotopological shifts and spectral barcodes

V. Distribution of surface species formed in $^{13}CO_2$-saturated water

VI. Analysis of photon flux

VII. Calculation of temperature rise resulting from photothermal heating

VIII. Supplementary movie captions

IX. Supplementary References

I. Sample preparation for surface-enhanced Raman scattering (SERS) experiments

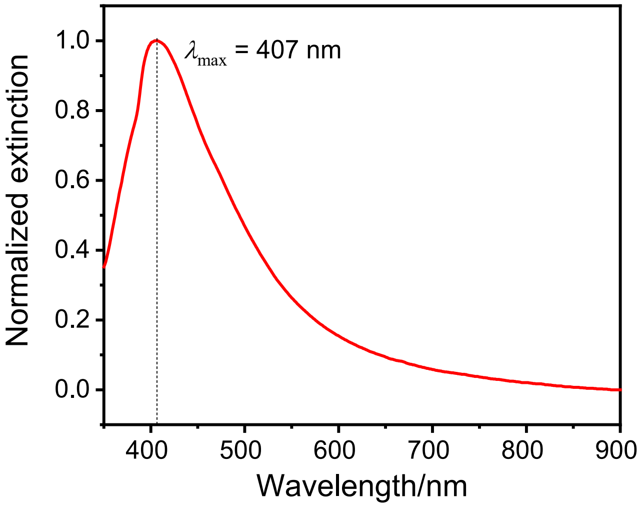

Supplementary Figure 1. Extinction spectrum (normalized to the maximum extinction) of the Ag NP colloid synthesized by the Lee–Meisel method.[1] The spectrum shows the localized surface plasmon resonance (LSPR) band with a wavelength maximum, λ_{max}, at 407 nm (marked by the dotted vertical line).

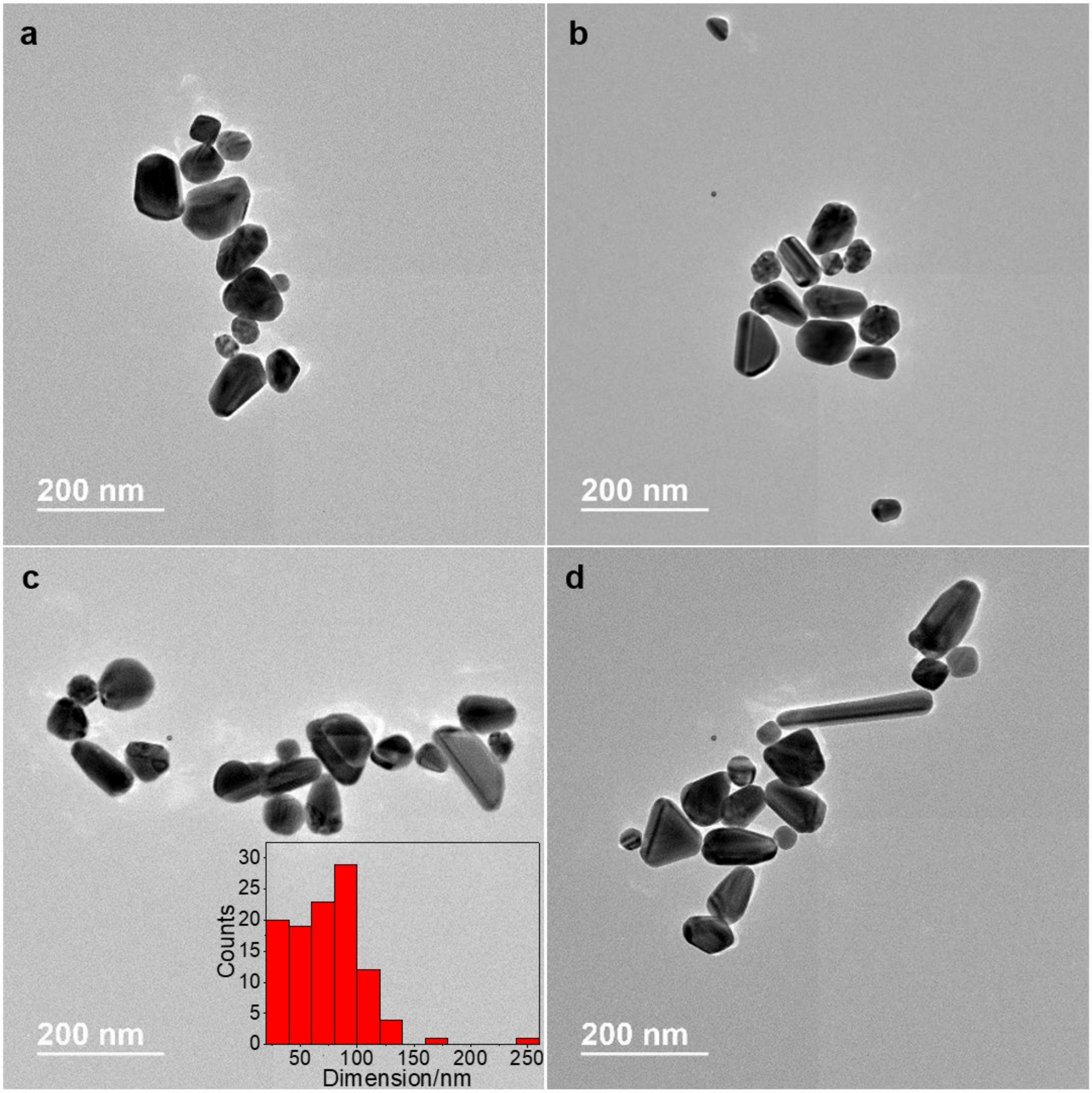

Supplementary Figure 2. Transmission electron microscopy (TEM) characterization of colloidal Ag NPs synthesized by the Lee–Meisel method. A 500 µL volume of the as-synthesized colloid was mixed with 3 mL of DI water. The diluted colloid was drop-casted onto a carbon-coated Formvar film on a 200 mesh Cu TEM grid (Ted Pella) and dried by heating on a hot plate for < 5 min. Images were acquired on a JEOL 2100 Cryo TEM instrument using a 200 kV electron beam. (a–d) Selected TEM images show that the colloid contains NPs of a variety of sizes and shapes. From an analysis of 109 NPs over several images, the longest NP dimension is found to range from 26–244 nm with an average of 74 nm and a standard deviation of 33 nm. The histogram of longest NP dimension is shown in the inset in c.

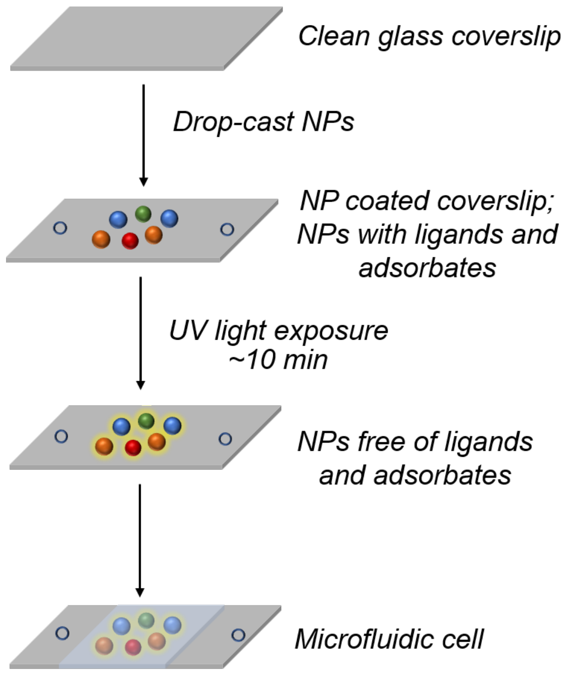

Supplementary Figure 3. Schematic description of the construction of microfluidic reaction cells for SERS experiments.

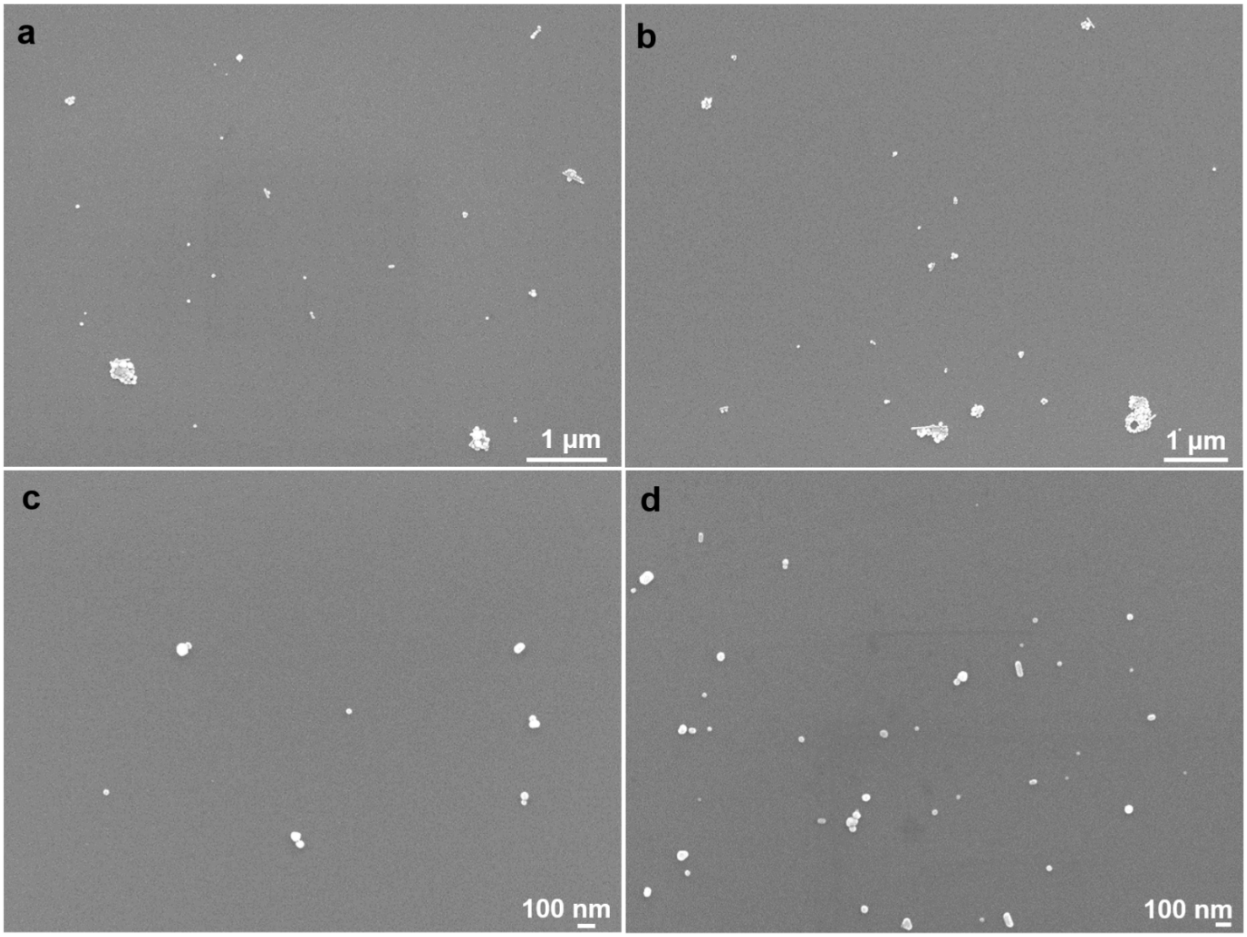

Supplementary Figure 4. Scanning electron microscopy (SEM) characterization of Ag NPs deposited on a substrate using conditions similar to the preparation of samples for the in-situ SERS studies. A 10 μL volume of the as-synthesized Ag NP colloid was mixed with 1 mL of DI water. The diluted colloid was drop-casted onto a Si substrate and dried by heating on a hot plate for < 5 min. Imaging was performed using a JEOL 7000F field emission SEM instrument operated in secondary-electron mode. An accelerating voltage of 15 kV was used for imaging. (a–d) Selected images show the area distribution of discrete deposits formed by the sample preparation method used for our SERS studies. The area-density of NPs deposited is low enough such that discrete deposits well-isolated from one another are formed. The discrete deposits appear primarily to be single NPs, dimers, or trimers from the higher-magnification images in c and d. Some larger NP aggregates are found as well in the lower-magnification images in a and b.

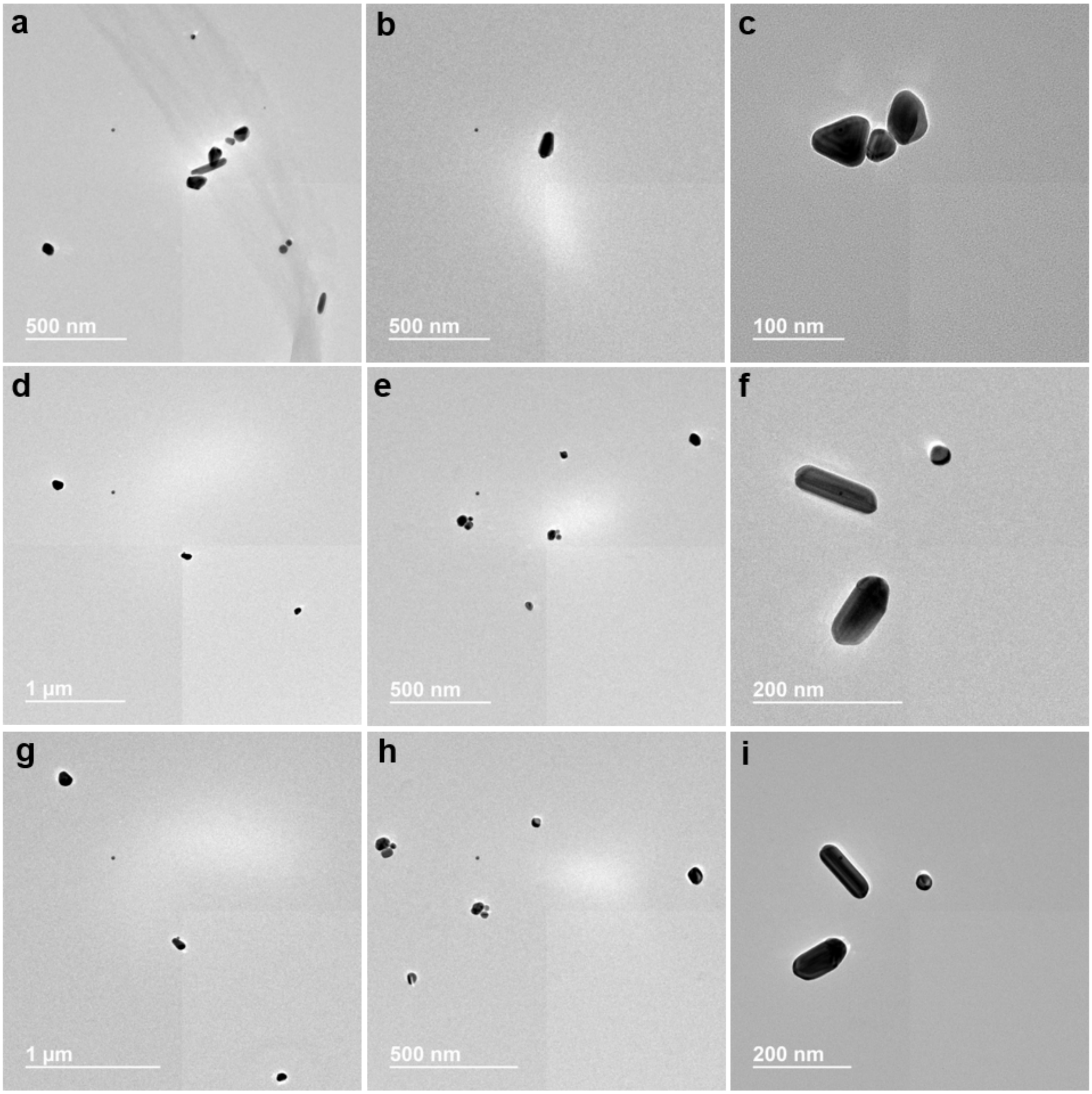

Supplementary Figure 5. TEM characterization of Ag NPs deposited on a substrate using conditions similar to the preparation of samples for the SERS studies. A 10 μL volume of the Ag NP colloid was mixed with 1 mL of DI water. The diluted colloid was drop-casted onto a carbon-coated Formvar film on a 200 mesh, Cu TEM grid (Ted Pella) and dried by heating on a hot plate for < 5 min. Images at multiple magnifications were acquired on a JEOL 2100 Cryo TEM instrument using a 200 kV electron beam. (a–i) Selected TEM images show the area distribution of discrete deposits formed by the sample preparation method used for our SERS studies. Similar to the findings from SEM shown in Supplementary Fig. 4, these TEM images show discrete deposits well-isolated from one another and consisting primarily of single NPs, dimers, or trimers.

Some larger aggregates are also present in other regions. The same regions seen in the images in d–f were imaged after subjecting the TEM grid-bearing sample to ~10 min of UV light illumination. The respective images in g–i show that UV light illumination does not induce changes in the area-density or location of deposited NPs.

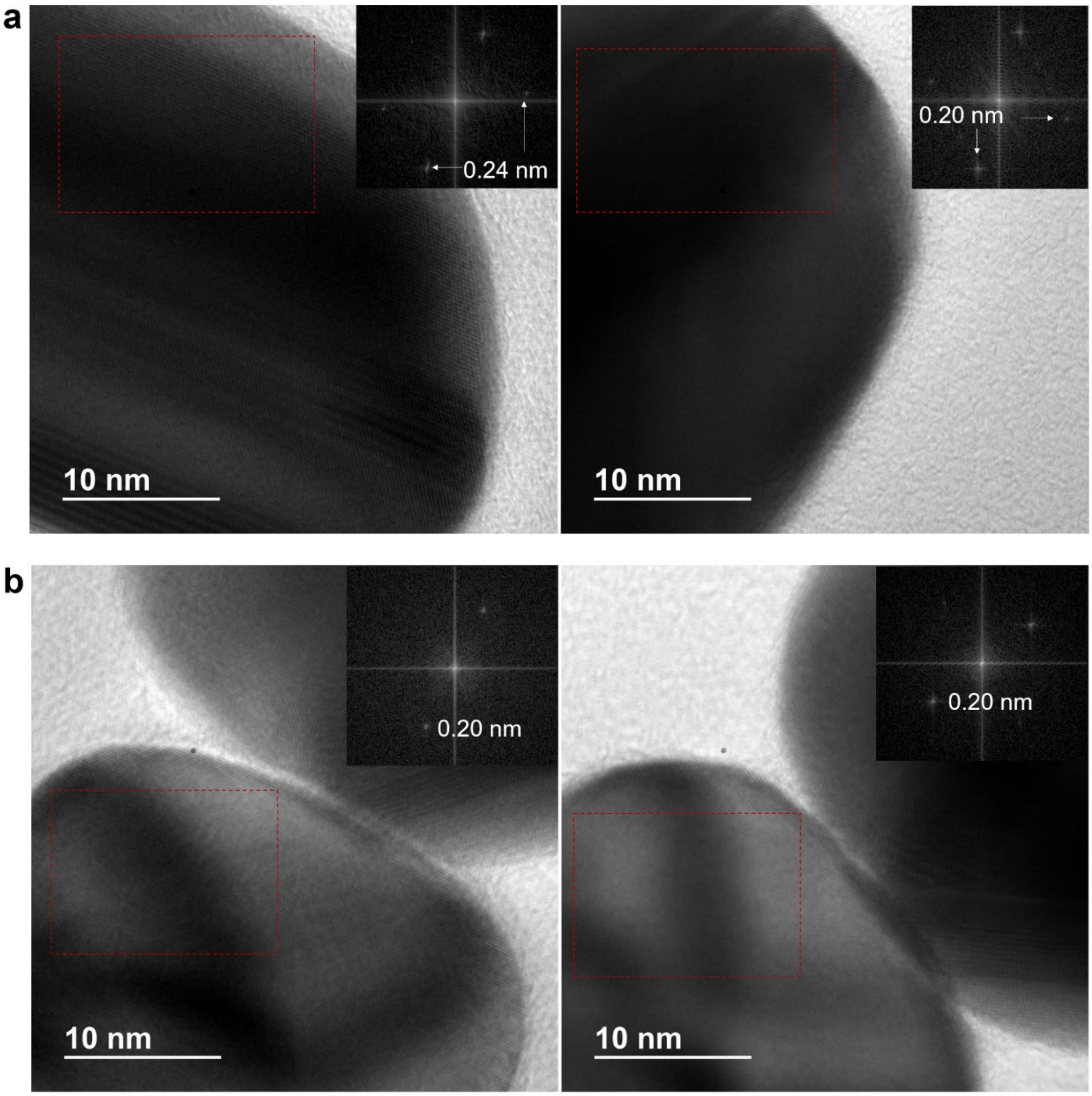

a
0.24 nm
10 nm
0.20 nm
10 nm
b
0.20 nm
10 nm
0.20 nm
10 nm

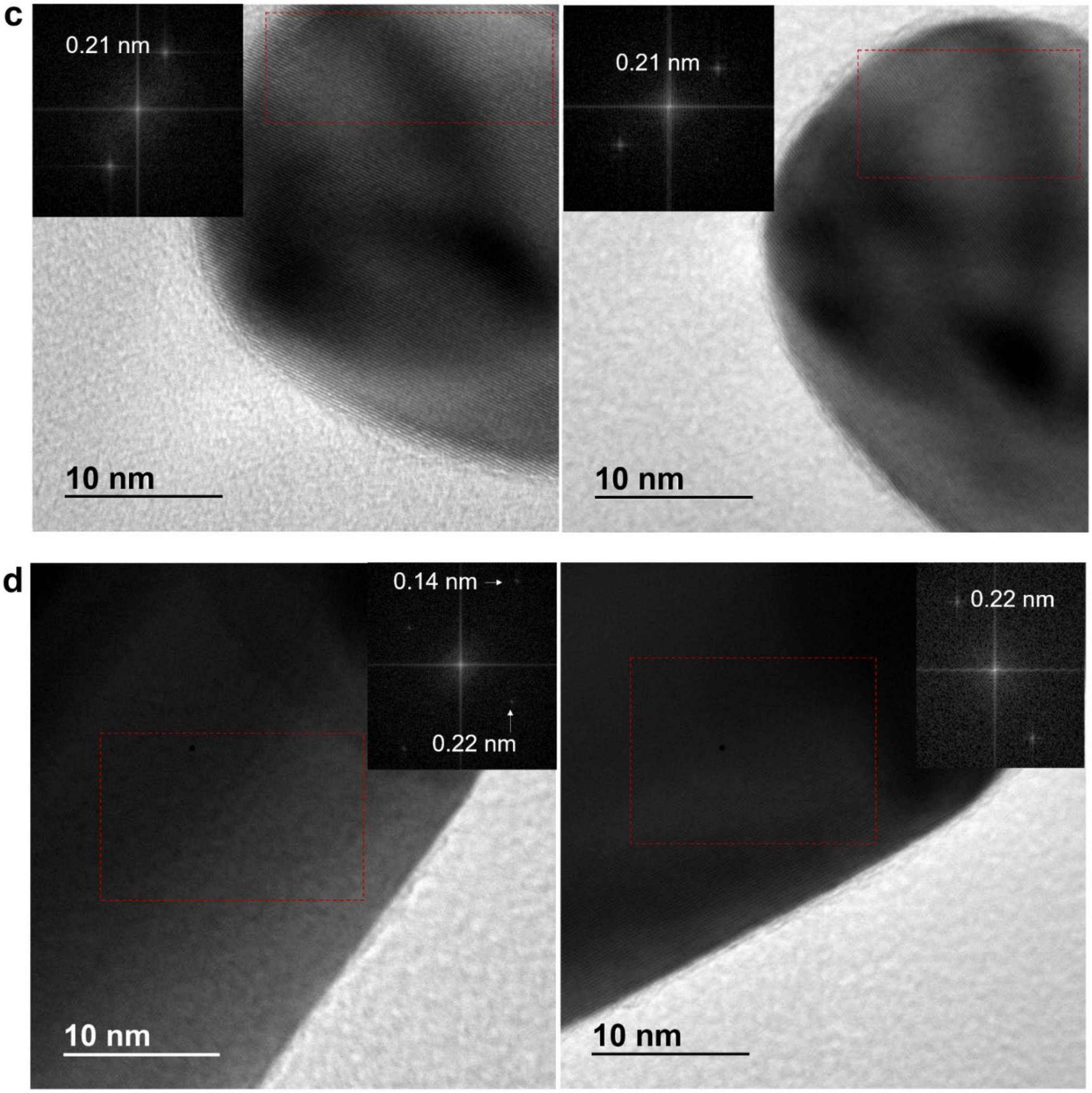

Supplementary Figure 6. High-magnification TEM images of representative NPs before (left column) and after (right column) ~10 min of UV light illumination. In each image, a Fast-Fourier transform (FFT) of a selected area (red box) of the real-space image is shown as the inset. Reciprocal lattice spots at a d-spacing of 0.14 nm (inset of d) correspond to (220) lattice planes of Ag. Reciprocal lattice spots at d-spacings of 0.22 nm (inset of d) and 0.24 nm (inset figure a) correspond to the (111) lattice planes of Ag and those at 0.20 nm (insets in a & b) and 0.21 nm (inset figure c) correspond to the (200) lattice planes of Ag. No oxide shell is found on the Ag NPs subjected to UV light illumination. The morphology of the NPs does not appear to have been modified by UV light illumination. The high-magnification images shown here are from the same study as the one presented in Supplementary Fig. 5. Note the display of each FFT image has been adjusted in ImageJ by setting the brightness/contrast minimum to 100.

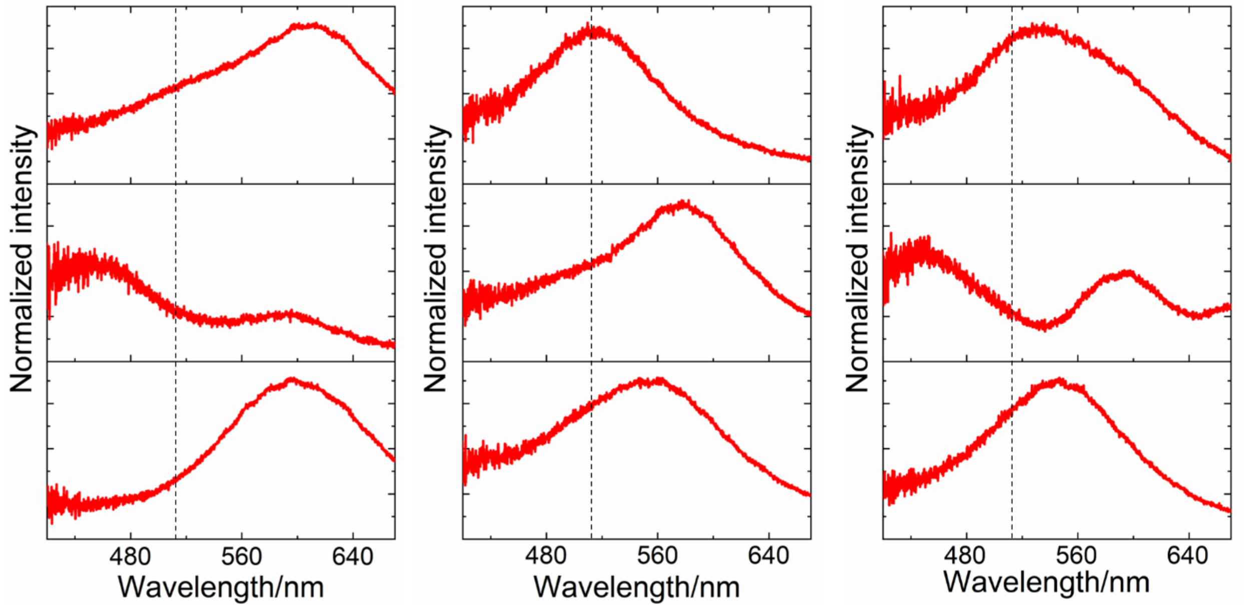

Supplementary Figure 7. Representative dark-field scattering spectra of discrete Ag NP scatterers on a sample prepared using conditions similar to the preparation of samples for the in-situ SERS studies. The scattering spectra of these NP scatterers show broad LSPR peaks centered at wavelengths in the 450–600 nm range. Thus, representative scatterers interrogated in our study possess LSPR bands overlapping with the 514.5 nm laser excitation (vertical dotted line). The scattering spectra also indicate that the scatterers are not single spherical NPs, which would exhibit a simple dipolar LSPR band centered around 400 nm. Instead, a typical scatterer interrogated in our study is an anisotropic NP, a NP dimer, or a few-NP aggregate, as further supported by SEM (Supplementary Fig. 4) and TEM (Supplementary Fig. 5) characterization of substrate-bound Ag NPs prepared using conditions similar to those used for preparation of samples for SERS studies. The spectra of discrete scatterers were measured using a dark-field microscopy setup under white-light illumination focused using a dark-field (Olympus U-DCW) condenser. The scattered light was collected by a 100× dark-field, oil-immersion objective, dispersed by a spectrograph equipped with a 300 g/mm grating, and spectrally analyzed using a Pylon 100B charged-coupled device (CCD) camera. Acquired spectra were subjected to a subtraction of the dark counts and divided by the dark-count-subtracted spectrum of the white-light source to obtain a scattering spectrum. The scattering spectra were normalized to a [0, 1] range prior to plotting.

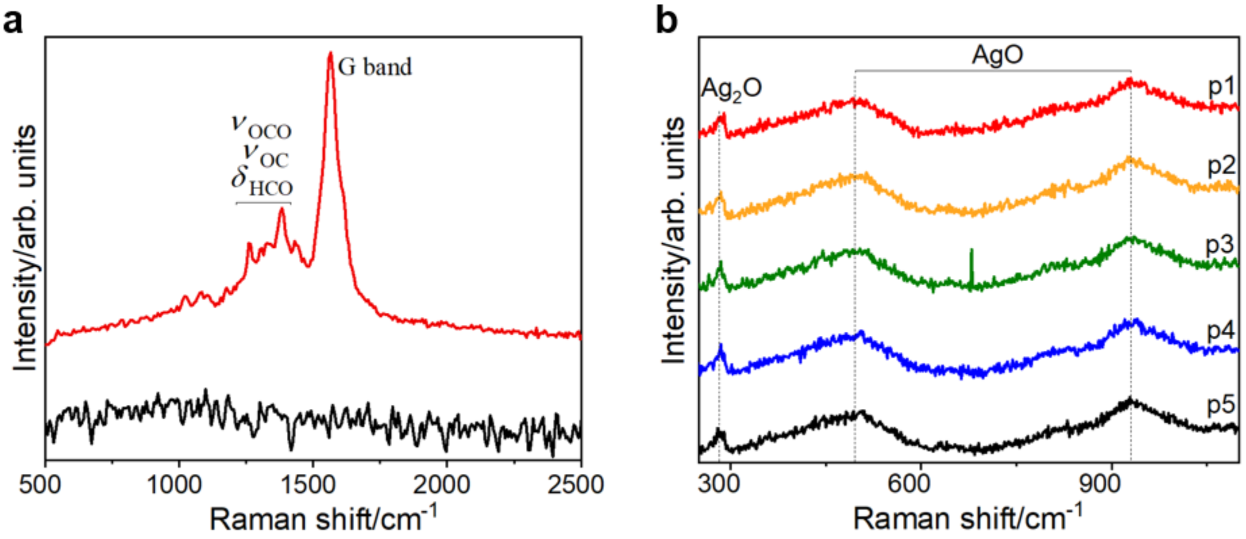

Supplementary Figure 8. a) A representative SERS spectrum of a Ag NP scatterer before (red trace) and after 10 min of UV light illumination (black trace). The spectrum before illumination shows SERS bands corresponding to citrate ligands (as shown by the labeled modes where ν refers to stretching and δ to bending) and adsorbed carbonaceous species (as shown by the labeled G band) on the NP; no bands are observed in the spectrum acquired after 10 min of illumination. The spectra were collected under 9 mW of 514.5 nm excitation with an acquisition time of 1 s (red trace) and 200 ms (black trace). Spectra were normalized to a [0,1] scale and the black trace was smoothed using a Savitzky–Golay method filter with a window of 5 points and a polynomial of order 3 prior to plotting in a vertically-stacked manner. b) SERS spectra in the low-wavenumber range (250-1100 cm^{-1}) acquired from five discrete scatterers (labeled p1 to p5) on a Ag NP-coated glass coverslip that was subjected to 10 min of UV light illumination. The spectra were collected under 20 mW of 514.5 nm excitation with an acquisition time of 1 min. The vibrational peak (vertical dotted line) at 284 cm^{-1} can be attributed to the presence of AgO.[2] Broad vibrational peaks (vertical dotted lines) centered at 497 cm^{-1} and 930 cm^{-1} can be assigned to Ag_2O.[2, 3] SERS spectra were baseline-subtracted prior to plotting in a vertically-stacked manner. The baseline was determined by polynomial fitting.

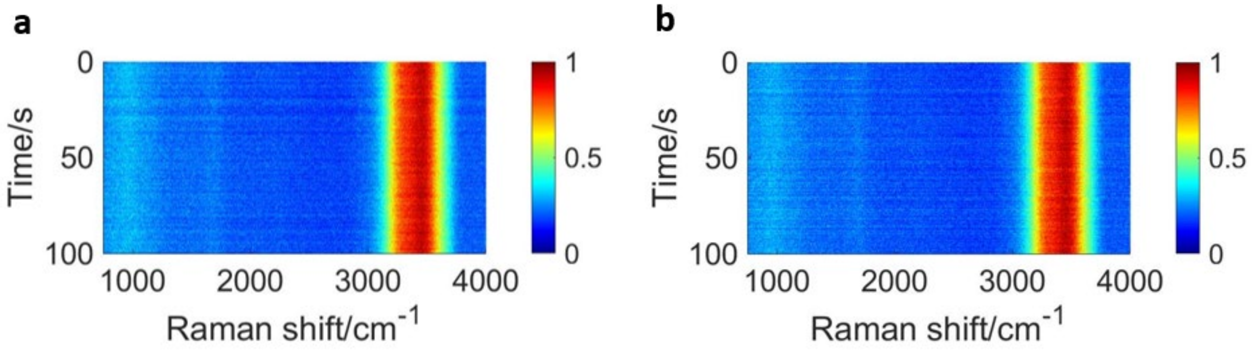

Supplementary Figure 9. Two examples (a and b) of spectrograms showing the time series of in-situ SERS spectra (normalized to a [0, 1] scale at each time-point) acquired from an individual Ag NP scatterer in CO_2-untreated $H_2O_{(l)}$ under plasmonic excitation. The SERS intensity is coded by the color, as indicated by the legend. Except for the broad band at ca. 3400 cm^{-1}, which is due to Raman scattering from $H_2O_{(l)}$ in the medium, there are no SERS bands in the spectra. This shows the cleanliness of the NPs.

Statistics showing the cleanliness of Ag NPs after UV light illumination

<u>Cleanliness of individual scatterers</u>

- Total number of individual scatterers investigated = 105
- Number of scatterers with at least one unclean spectral frame = 12
- Number of scatterers with no unclean spectral frames = 93.
- Percentage of such fully clean scatterers = 89 %

<u>Cleanliness of spectral frames</u>

- Total number of spectral frames investigated = 105 scatterers x 500 frames = 52,500 frames
- Number of unclean frames = 738
- Percentage of clean frames = 98.6 %

<u>Degree of cleanliness of scatterers with at least one unclean frame</u>

- Total number of frames acquired from scatterers with at least one unclean frame = 12 scatterers × 500 = 6,000 frames
- Number of unclean frames = 738
- Percentage of clean frames acquired from scatterers with at least one unclean frame = 88 %

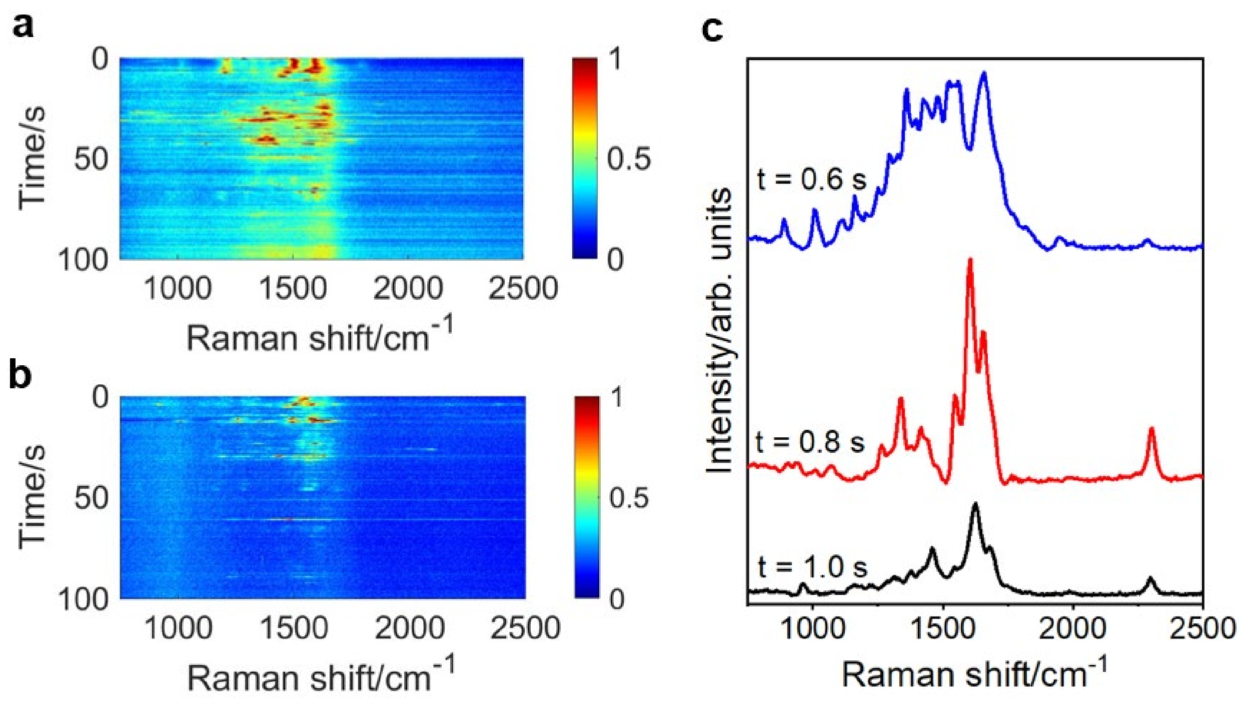

Supplementary Figure 10. Two representative examples (a and b) of spectrograms showing the time series of in-situ SERS spectra (normalized to a [0, 1] scale at each time-point) acquired from an individual Ag NP scatterer in CO_2-saturated water under plasmonic excitation. The SERS intensity is coded by the color, as indicated by the legend. c) SERS spectra presented in Fig. 1d are plotted here without normalization to demonstrate the intensity variations from one time-frame to another. The spectra were baseline-subtracted prior to plotting in a vertically-stacked manner. The baseline was determined by polynomial fitting.

II. Reference database and modes for the assignment of SERS spectra to species

Supplementary Table 1. Database of chemical species used for assignment of SERS spectra

Category	Chemical species
C_1	carbon monoxide
	bicarbonate
	methane
	formic acid
	formaldehyde
	methanol
C_2	glyoxal
	ethylene
	oxalic acid
	acetaldehyde
	acetic acid
	ethanol
	glycolaldehyde
	ethane
	ethylene glycol
C_3	acetone
	propene
	allyl alcohol
	propane
	hydroxyacetone
	propionaldehyde
	propanol
C_4	butanol

Potential energy distribution (PED) analysis: The interpretation of DFT-computed Raman spectra and the assignment of vibrational frequencies to various local bond vibrations were performed by a PED analysis. This analysis was performed using the vibrational energy distribution analysis (VEDA) program.[4, 5] A normal vibrational mode of a polyatomic molecule is typically a superposition of multiple local stretching, bending, and torsion motions of smaller fragments of the molecule. PED analysis helps us quantitatively assess the contributions of these local motions to a normal mode of vibration. In this analysis, the co-ordinate of a normal mode of vibration is represented as a superposition of various local mode co-ordinates. The VEDA program uses output files from Gaussian and proposes an introductory set of possible local mode co-ordinates comprising a normal mode of vibration. The combination of local mode co-ordinates is then optimized by the program. VEDA outputs the % contribution of a local mode co-ordinate to the potential energy function of a normal mode. In the following tables (Supplementary Tables 2–24), alongside each vibrational frequency, the dominant local mode/s and their % PED contribution to the frequency are listed in the second column. Positive values indicate symmetric modes and negative values indicate antisymmetric modes of vibration. The following symbols are used to indicate various vibrational modes: ν-stretching; δ-bending; ρ-rocking; τ-twisting; ω-wagging; ϕ-torsion.

Supplementary Table 2. DFT-computed Raman frequencies and normal mode analysis for formaldehyde.

formaldehyde		
$^{12}C\ \upsilon$ in cm^{-1}	Major vibrational component (PED %)	$^{13}C\ \upsilon$ in cm^{-1}
1201	ϕ_{out} CHOH (100)	1189
1259	δ HCO (100)	1249
1530	δ HCH (90)	1530
1813	v OC (90)	1773
2889	v CH (100)	2885
2948	v CH (100)	2935

Supplementary Table 3. DFT-computed Raman frequencies and normal mode analysis for formic acid

formic acid		
$^{12}C\ \upsilon$ in cm^{-1}	Major vibrational component (PED %)	$^{13}C\ \upsilon$ in cm^{-1}
1050	ϕ_{out} COOH (-100)	1009
1128	v OC (78)	1116
1266	δ HOC (76)	1276
1421	δ HCO (92)	1402
1792	v OC (87)	1763

Supplementary Table 4. DFT-computed Raman frequencies and normal mode analysis for bicarbonate

bicarbonate		
$^{12}C\ \upsilon$ in cm^{-1}	Major vibrational component (PED %)	$^{13}C\ \upsilon$ in cm^{-1}
557	δ OCO (87)	555
1030	v OC (88)	1027
1149	v OC (-59)	1136
1269	δ HOC (64)	1252
1567	v OC (93)	1527

Supplementary Table 5. DFT-computed Raman frequencies and normal mode analysis for methane

**methane**		
^{12}C υ in cm^{-1}	Major vibrational component (PED %)	^{13}C υ in cm^{-1}
1340	δ HCH (70); ϕ_{out} CHHH (-93); ϕ_{out} CHHH (-69)	1331
1558	δ HCH (98); δ HCH (98)	1558
3026	ν CH (100)	3026
3132	ν CH (91); ν CH (91); ν CH (85)	3121

Supplementary Table 6. DFT-computed Raman frequencies and normal mode analysis for methanol

**methanol**		
^{12}C υ in cm^{-1}	Major vibrational component (PED %)	^{13}C υ in cm^{-1}
1040	ν OC (71)	1024
1070	ϕ_{out} CHOH (37); δ HOC (-30)	1064
1168	ϕ_{out} CHOH (-85)	1160
1357	δ HOC (58)	1349
1479	δ HCH (92)	1473
1494	δ HCH (85)	1492
1505	δ HCH (-82)	1503

Supplementary Table 7. DFT-computed Raman frequencies and normal mode analysis for carbon monoxide

**carbon monoxide**		
^{12}C υ in cm^{-1}	Major vibrational component (PED %)	^{13}C υ in cm^{-1}
2213	ν OC	2163

Supplementary Table 8. DFT-computed Raman frequencies and normal mode analysis for ethane

ethane		
^{12}C υ in cm^{-1}	Major vibrational component (PED %)	^{13}C υ in cm^{-1}
996	ν CC (98)	965
1217	ϕ HCCH (-71);<br>ϕ_{out} CHCH (-43)	1201
1408	δ HCH (-81)	1398
1423	δ HCH (86)	1412
1503	δ HCH (76);<br>δ HCH (69)	1502
1506	δ HCH (-54);<br>δ HCH (77)	1503

Supplementary Table 9. DFT-computed Raman frequencies and normal mode analysis for ethanol

ethanol		
^{12}C υ in cm^{-1}	Major vibrational component (PED %)	^{13}C υ in cm^{-1}
897	ν OC (56)	882
1032	ν CC (65)	1010
1094	ν OC (30);<br>ϕ HCCO (27)	1077
1176	ϕ HCCO (-76)	1160
1262	δ HOC (58)	1254
1299	δ HCO (81)	1295
1404	δ HCH (77)	1394
1448	ϕ HCCH (-46)	1432
1481	δ HCH (72)	1479
1500	δ HCH (67)	1497
1526	δ HCH (-57)	1521

Supplementary Table 10. DFT-computed Raman frequencies and normal mode analysis for ethylene

**ethylene**		
^{12}C υ in cm^{-1}	Major vibrational component (PED %)	^{13}C υ in cm^{-1}
977	ϕ HCCH (77)	969
1058	ϕ HCCH (100)	1058
1238	δ HCC (-100)	1220
1378	δ HCH (65)	1365
1472	δ HCH (100)	1466
1684	v CC (65)	1637

Supplementary Table 11. DFT-computed Raman frequencies and normal mode analysis for ethylene glycol

**ethylene glycol**		
^{12}C υ in cm^{-1}	Major vibrational component (PED %)	^{13}C υ in cm^{-1}
871	ϕ HCCO (63)	871
1034	v OC (63)	1017
1045	δ HOC (27);<br>ϕ HCCO (19)	1030
1096	v CC (-70)	1073
1183	δ HOC (37);<br>δ HCO (37)	1177
1243	δ HOC (-28);<br>δ HCCO (-20)	1232
1376	δ HOC (44)	1370
1378	δ HOC (-42)	1374
1396	ϕ HCCO (54)	1387
1411	δ HCH (74)	1398
1486	δ HCH (71)	1482
1491	δ HCH (-77)	1487

Supplementary Table 12. DFT-computed Raman frequencies and normal mode analysis for acetic acid

acetic acid		
^{12}C υ in cm^{-1}	Major vibrational component (PED %)	^{13}C υ in cm^{-1}
999	ϕ HCCO (49)	989
1067	ϕ HCCO (-58)	1048
1205	ν CC (-27); δ HOC (28)	1190
1332	δ HOC (-52)	1313
1408	δ HCH (42)	1392
1472	δ HCH (41)	1470
1476	δ HCH (75)	1474
1818	ν OC (83)	1773

Supplementary Table 13. DFT-computed Raman frequencies and normal mode analysis for acetaldehyde

acetaldehyde		
^{12}C υ in cm^{-1}	Major vibrational component (PED %)	^{13}C υ in cm^{-1}
886	ν_s CC (63)	868
1128	ϕ HCCO (-40); ν_{as} CC (-23)	1104
1133	ϕ HCCO (76)	1113
1377	δ HCH (88)	1366
1420	δ HCO (85)	1415
1460	δ HCH (-71)	1458
1469	δ HCH (-76)	1467
1808	ν_s OC (90)	1765
2871	ν_s CH (99)	2863

Supplementary Table 14. DFT-computed Raman frequencies and normal mode analysis for oxalic acid

**oxalic acid**		
^{12}C υ in cm^{-1}	Major vibrational component (PED %)	^{13}C υ in cm^{-1}
839	ϕ_{out} OCOC (98)	810
1139	ν OC (69)	1127
1207	δ HOC (58)	1198
1325	δ HOC (63)	1308
1399	ν CC (47);<br>δ HOC (27)	1364
1826	ν OC (93)	1783
1836	ν OC (85)	1789

Supplementary Table 15. DFT-computed Raman frequencies and normal mode analysis for glycolaldehyde

**glycolaldehyde**		
^{12}C υ in cm^{-1}	Major vibrational component (PED %)	^{13}C υ in cm^{-1}
852	ν CC (74)	832
1100	ϕ OCCO (-69)	1081
1131	ν OC (73)	1108
1212	δ HOC (73)	1209
1257	δ HCO (75)	1254
1411	δ HCH (-80)	1405
1428	δ HCH (69)	1412
1480	ϕ HCCO (73)	1474
1831	ν OC (91)	1788

Supplementary Table 16. DFT-computed Raman frequencies and normal mode analysis for glyoxal

**glyoxal**		
^{12}C υ in cm^{-1}	Major vibrational component (PED %)	^{13}C υ in cm^{-1}
810	ϕ HCCO (77)	805
1059	ν CC (-49);<br>δ CCO (46)	1024
1072	ϕ HCCO (100)	1052
1334	δ HCO (98)	1328
1376	δ HCO (88)	1373
1798	ν OC (99)	1758
1801	ν OC (88)	1760

Supplementary Table 17. DFT-computed Raman frequencies and normal mode analysis for propane

**propane**		
^{12}C υ in cm^{-1}	Major vibrational component (PED %)	^{13}C υ in cm^{-1}
870	v CC (85)	846
914	ϕ HCCC (57)	910
932	ϕ HCCC (67)	925
1056	v CC (-87)	1028
1174	ϕ HCCC (55)	1152
1212	ϕ HCCC (-75)	1191
1317	δ HCC (55)	1311
1367	ϕ HCCH (-76)	1351
1404	δ HCH (79)	1393
1421	δ HCH (81)	1410
1490	δ HCH (-57)	1488
1492	δ HCH (-79)	1490
1497	δ HCH (57)	1495
1507	δ HCH (75)	1505
1513	δ HCH (80)	1509

Supplementary Table 18. DFT-computed Raman frequencies and normal mode analysis for propanol

propanol		
^{12}C υ in cm^{-1}	Major vibrational component (PED %)	^{13}C υ in cm^{-1}
860	*v* CC (76)	838
926	ϕ HCCC (57)	919
979	*v* OC (47)	966
1061	*v* CC (-64)	1036
1109	*v* OC (-31); ϕ HCCC (17)	1091
1156	ϕ HCCC (-37); ϕ OCCC (16)	1136
1239	δ HOC (47)	1230
1271	δ HCO (56)	1265
1322	δ HCC (42)	1316
1380	ϕ HCCO (-54)	1365
1410	δ HCH (79)	1398
1444	ϕ HCCC (50)	1429
1478	δ HCH (84)	1474
1495	δ HCH (-65)	1493
1509	δ HCH (-64)	1506
1518	δ HCH (67)	1514

Supplementary Table 19. DFT-computed Raman frequencies and normal mode analysis for propene

propene		
^{12}C υ in cm^{-1}	Major vibrational component (PED %)	^{13}C υ in cm^{-1}
948	ϕ HCCC (41); δ HCC (39)	943
1025	ϕ HCCH (85)	1023
1070	ϕ HCCC (61)	1055
1189	ϕ HCCC (-27); δ HCC (25)	1166
1326	δ HCC (70)	1315
1407	δ HCH (90)	1397
1448	δ HCH (69)	1437
1480	δ HCH (-77)	1477
1494	δ HCH (-64)	1491
1704	*v* CC (68)	1649

Supplementary Table 20. DFT-computed Raman frequencies and normal mode analysis for propionaldehyde

**propionaldehyde**		
^{12}C υ in cm^{-1}	Major vibrational component (PED %)	^{13}C υ in cm^{-1}
905	ϕ HCCC (59)	900
1004	v CC (42);<br>ϕ HCCC (38)	990
1108	ϕ HCCC (27);<br>v CC (-25)	1082
1144	ϕ HCCC (43)	1123
1279	δ HCC (51)	1271
1362	ϕ HCCC (-35);<br>δ HCH (-22)	1339
1408	δ HCH (-82)	1401
1422	δ HCH (74)	1415
1449	δ HCH (70)	1443
1494	δ HCH (75)	1492
1501	δ HCH (-64)	1498
1803	v OC (89)	1761

Supplementary Table 21. DFT-computed Raman frequencies and normal mode analysis for acetone

**acetone**		
^{12}C υ in cm^{-1}	Major vibrational component (PED %)	^{13}C υ in cm^{-1}
889	ϕ HCCC (-48)	877
1083	ϕ HCCC (-64)	1071
1116	ϕ HCCC (33);<br>ϕ_{out} OCCC (-32)	1093
1232	v CC (38)	1198
1386	δ HCH (-82)	1372
1387	δ HCH (93)	1375
1461	δ HCH (-73)	1459
1465	δ HCH (-74)	1462
1472	δ HCH (-73)	1469
1488	δ HCH (70)	1485
1786	v OC (90)	1743

Supplementary Table 22. DFT-computed Raman frequencies and normal mode analysis for hydroxyacetone

**hydroxyacetone**		
^{12}C υ in cm^{-1}	Major vibrational component (PED %)	^{13}C υ in cm^{-1}
856	ϕ HCCC (64)	847
983	ϕ HCCC (-34);<br>v CC (31)	966
1090	ϕ HCCC (47)	1068
1112	v OC (71)	1092
1199	v CC (16);<br>v CC (-12);<br>ϕ HCCC (15);<br>ϕ HCCC (-15)	1174
1251	δ HCO (71)	1247
1294	δ HOC (45)	1280
1391	δ HCH (89)	1379
1432	δ HCH (45)	1419
1466	δ HCH (-63)	1463
1477	ϕ HCCC (35);<br>δ HCH (-33)	1473
1480	δ HCH (-39)	1477
1776	v OC (89)	1733

Supplementary Table 23. DFT-computed Raman frequencies and normal mode analysis for allyl alcohol

**allyl alcohol**		
^{12}C υ in cm^{-1}	Major vibrational component (PED %)	^{13}C υ in cm^{-1}
886	ν_s CC (71)	863
954	ϕ HCCC (98)	945
1004	δ HCC (46)	992
1018	ϕ HCCC (89)	1011
1038	ϕ HCCO (-55)	1024
1133	ν_s OC (48)	1113
1226	δ HOC (72)	1221
1257	δ HCO (89)	1256
1323	δ HCC (71)	1310
1429	ϕ HOCH (-41)	1418
1455	δ HCC (-34)	1440
1501	δ HCH (80)	1497
1708	ν_s CC (72)	1651

Supplementary Table 24. DFT-computed Raman frequencies and normal mode analysis for butanol

**butanol**		
^{12}C υ in cm^{-1}	Major vibrational component (PED %)	^{13}C υ in cm^{-1}
978	ϕ HCCC (11);<br>ϕ HCOH (-10);<br>ϕ HCCC (16);<br>ϕ HCCC (-22)	971
1033	ν_{as} OC (-65)	1006
1072	ν_s CC (75)	1048
1118	ν_{as} OC (-15);<br>δ HOC (-11);<br>ϕ HCCC (-20)	1101
1156	ϕ HCCC (-12);<br>ϕ HCCC (11);<br>ϕ HCCC (-16);<br>ϕ OCCC (-12)	1134
1231	δ HOC (33);<br>δ HCC (14);<br>ϕ HCCC (-13)	1220
1257	ϕ HCCC (37);<br>δ HCO (-14)	1250
1301	δ HOC (-20);<br>δ HCC (32)	1292
1332	δ HCC (38);<br>ϕ HCCC (-12);	1323
1340	δ HCC (34)	1334
1403	ϕ HCCC (-50)	1384
1414	δ HCH (92)	1404
1443	δ HCO (39)	1428
1476	δ HCH (76)	1472
1496	δ HCH (-68)	1494
1501	δ HCH (73)	1499
1510	δ HCH (75)	1506
1516	δ HCH (81)	1512

III. SERS spectra of surface species detected

In Fig. 2, examples of measured SERS spectra corresponding to methanol, acetic acid, acetone, and butanol are provided. Representative examples of measured SERS spectra for the other species detected in plasmon-excitation-driven CO_2RR are shown below in Supplementary Figs. 11–19. These examples were found from 42,000 in-situ SERS spectra collected from 40 individual Ag NP scatterers in $^{12}CO_2$-saturated water under plasmonic excitation.

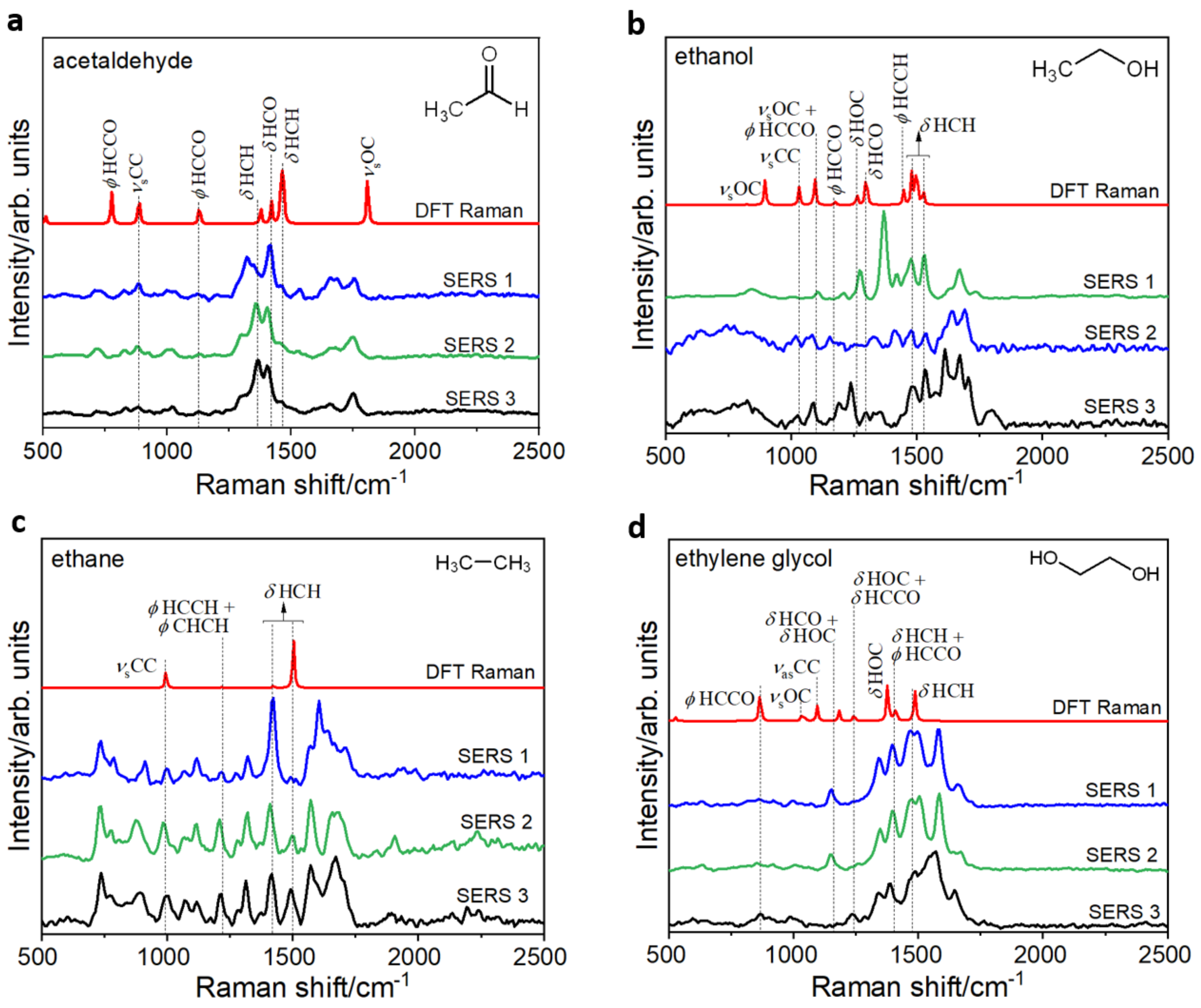

Supplementary Figure 11. Three representative examples of measured SERS spectra (blue, green, and black traces) that correspond to the detection of a) acetaldehyde, b) ethanol, c) ethane, and d) ethylene glycol. In each case, the DFT-computed Raman vibrational spectrum, which served as the basis of the assignment of the measured SERS spectra, is also shown by the red trace. The major vibrational modes are labeled using the following symbols: ϕ-torsion; ν_s-symmetric stretching; ν_{as}-asymmetric stretching; δ-bending. Spectra are shown vertically stacked for clarity. SERS spectra were baseline-subtracted, subjected to smoothing by the Savitzsky–Golay method with a window of 5 points and a polynomial of order 3, and normalized to a [0, 1] scale prior to plotting. The baseline was determined by polynomial fitting. DFT-computed Raman spectra were normalized to a [0, 1] scale, and the y-axis quantity was magnified (by a scale factor of 10 in panel a, a factor of 5 in panels b and d and a factor of 3 in panel c) prior to plotting.

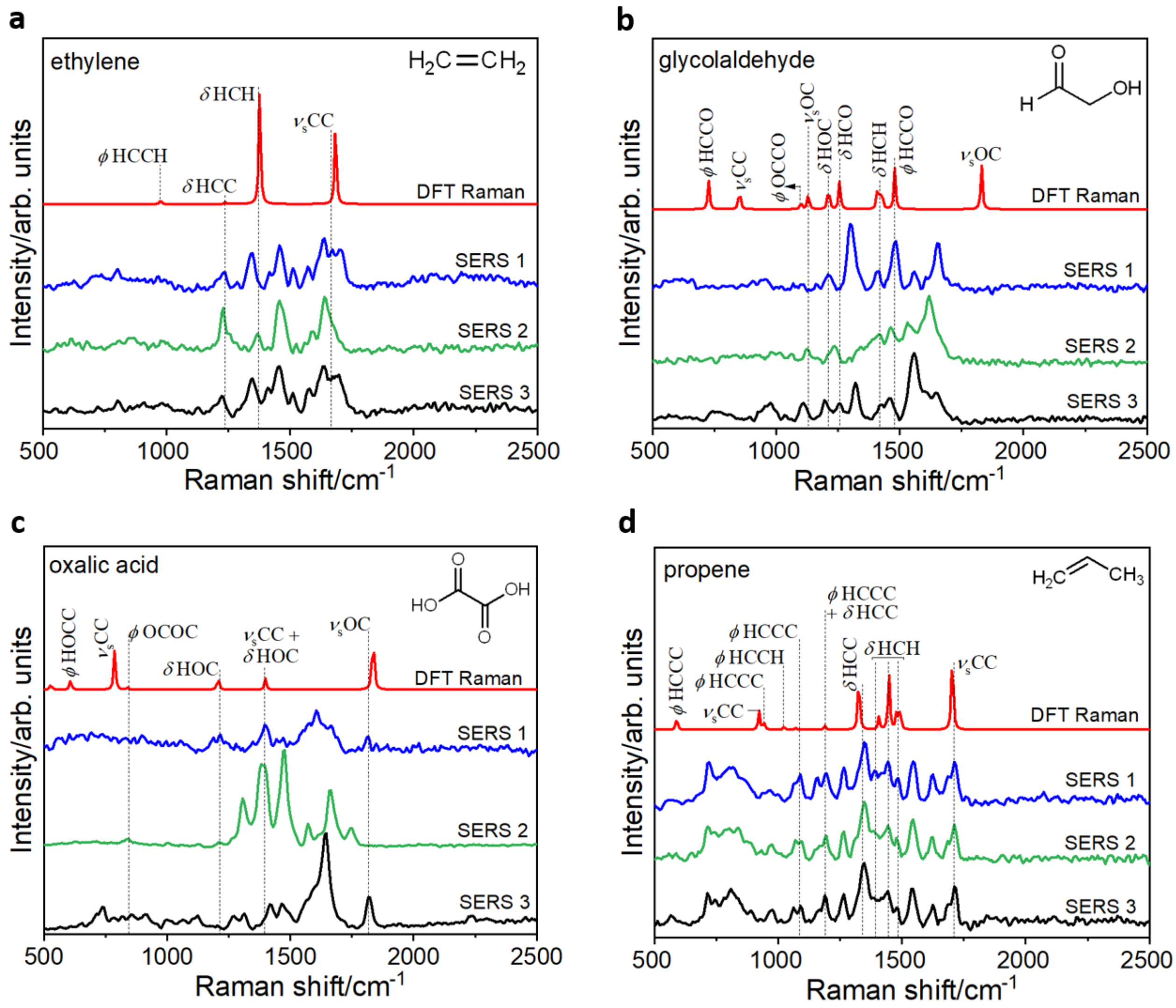

Supplementary Figure 12. Three representative examples of measured SERS spectra (blue, green, and black traces) that correspond to the detection of a) ethylene, b) glycolaldehyde, c) oxalic acid, and d) propene. In each case, the DFT-computed Raman vibrational spectrum, which served as the basis of the assignment of the measured SERS spectra, is also shown by the red trace. The major vibrational modes are labeled using the following symbols: ϕ-torsion; ν_s-symmetric stretching; ν_{as}-asymmetric stretching; δ-bending. Spectra are shown vertically stacked for clarity. SERS spectra were baseline-subtracted, subjected to smoothing by the Savitzky–Golay method with a window of 5 points and a polynomial of order 3, and normalized to a [0, 1] scale prior to plotting. The baseline was determined by polynomial fitting. DFT-computed Raman spectra were normalized to a [0, 1] scale, and the y-axis quantity was magnified (by a scale factor of 5 in panels a and b, a factor of 2 in panel c and a factor of 3 in panel d) prior to plotting.

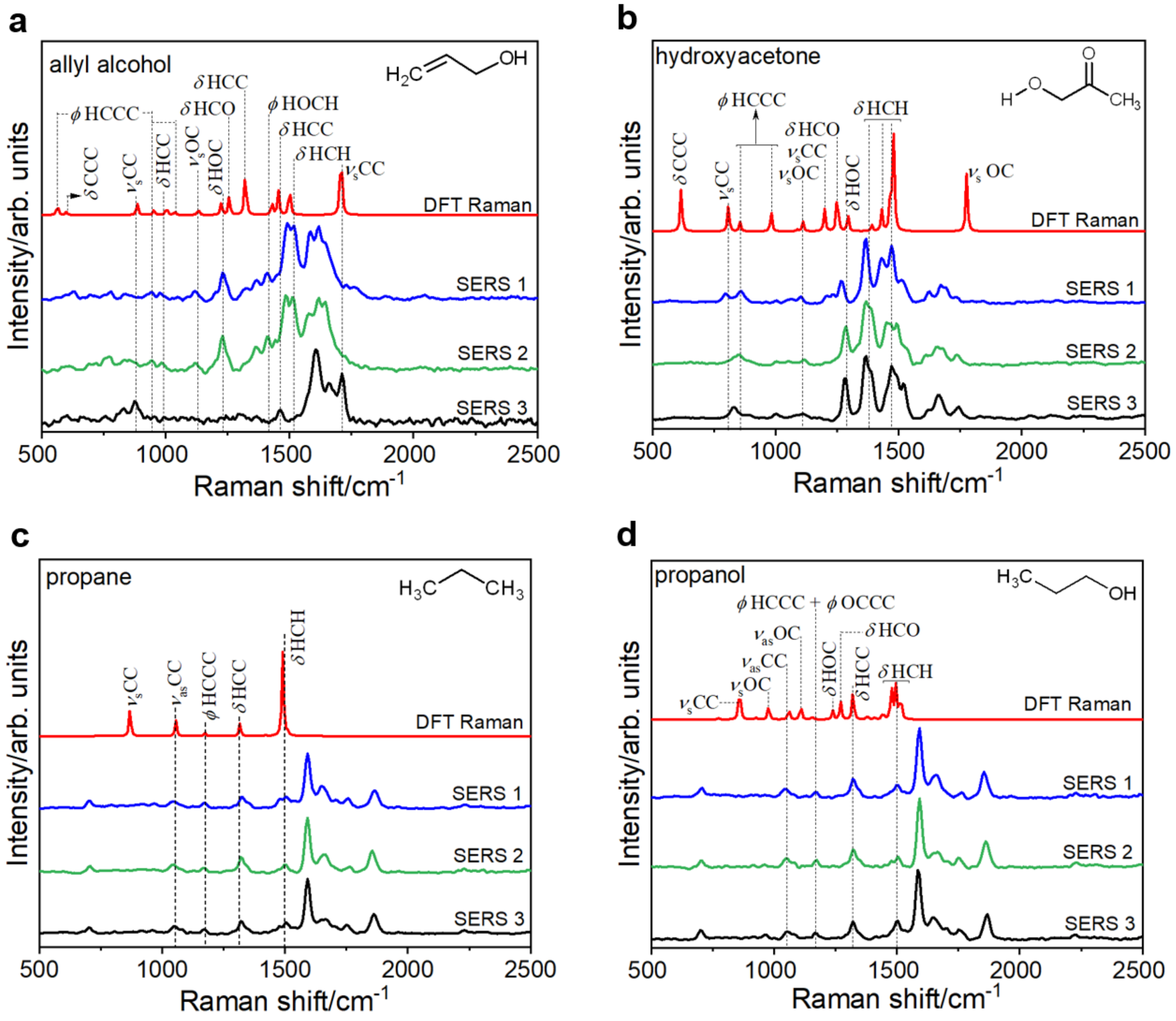

Supplementary Figure 13. Three representative examples of measured SERS spectra (blue, green, and black traces) that correspond to the detection of a) allyl alcohol, b) hydroxyacetone, c) propane, and d) propanol. In each case, the DFT-computed Raman vibrational spectrum, which served as the basis of the assignment of the measured SERS spectra, is also shown by the red trace. The major vibrational modes are labeled using the following symbols: ϕ-torsion; ν_s-symmetric stretching; ν_{as}-asymmetric stretching; δ-bending. Spectra are shown vertically stacked for clarity. SERS spectra were baseline-subtracted, subjected to smoothing by the Savitzsky–Golay method with a window of 5 points and a polynomial of order 3, and normalized to a [0, 1] scale prior to plotting. The baseline was determined by polynomial fitting. DFT-computed Raman spectra were normalized to a [0, 1] scale, and the y-axis quantity was magnified (by a scale factor of 3 in panel a and a factor of 10 in panels b, c and d) prior to plotting.

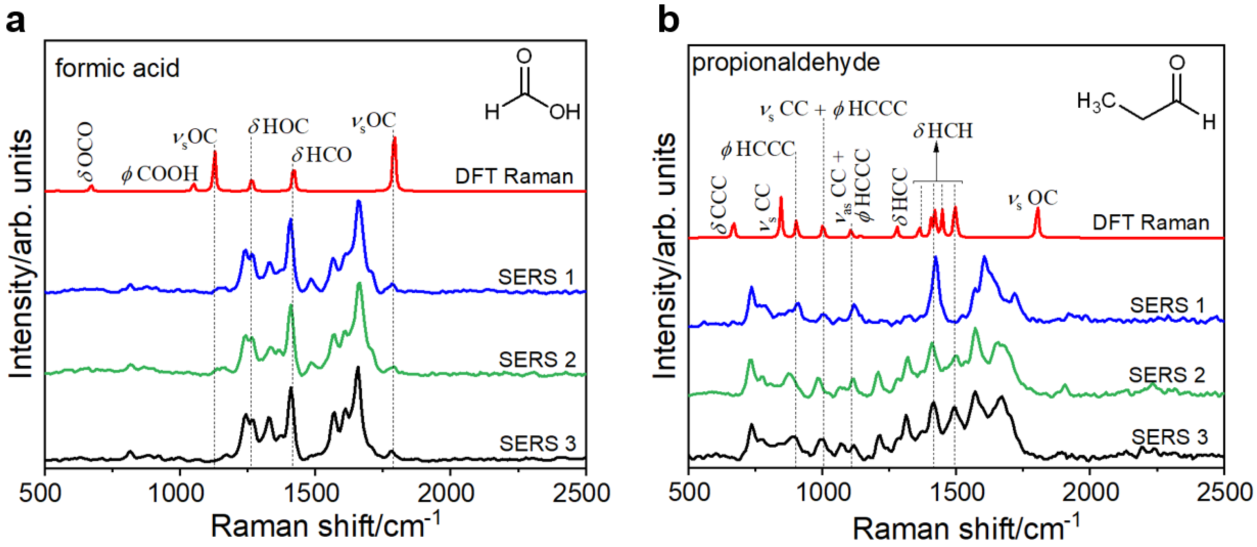

Supplementary Figure 14. Three representative examples of measured SERS spectra (blue, green, and black traces) that correspond to the detection of a) formic acid and b) propionaldehyde. In each case, the DFT-computed Raman vibrational spectrum, which served as the basis of the assignment of the measured SERS spectra, is also shown by the red trace. The major vibrational modes are labeled using the following symbols: ϕ-torsion; ν_s-symmetric stretching; ν_{as}-asymmetric stretching; δ-bending. Spectra are shown vertically stacked for clarity. SERS spectra were baseline-subtracted, subjected to smoothing by the Savitzsky–Golay method with a window of 5 points and a polynomial of order 3, and normalized to a [0, 1] scale prior to plotting. The baseline was determined by polynomial fitting. DFT-computed Raman spectra were normalized to a [0, 1] scale, and the y-axis quantity was magnified (by a scale factor of 4 in panel a and a factor of 5 in panel b) prior to plotting.

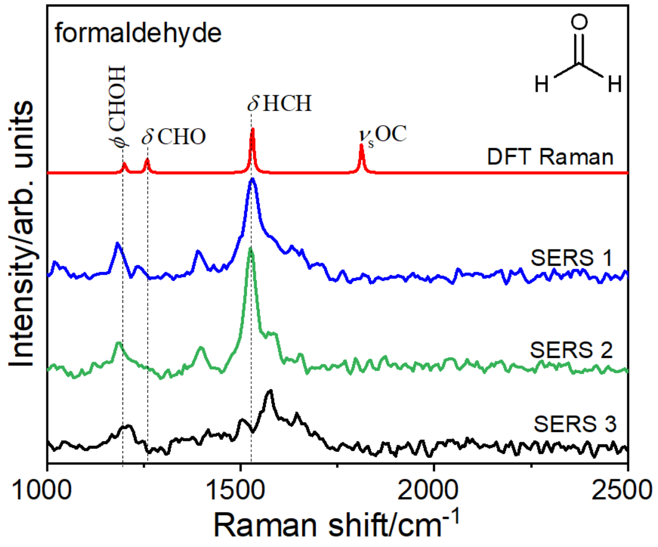

Supplementary Figure 15. Three representative examples of measured SERS spectra (blue, green, and black traces) that correspond to the detection of formaldehyde. The DFT-computed Raman vibrational spectrum, which served as the basis of the assignment of the measured SERS spectra, is also shown by the red trace. The major vibrational modes are labeled using the following symbols: ϕ-torsion; ν_s-symmetric stretching; ν_{as}-asymmetric stretching; δ-bending. Spectra are shown vertically stacked for clarity. SERS spectra were baseline-subtracted, subjected to smoothing by the Savitzsky–Golay method with a window of 5 points and a polynomial of order 3, and normalized to a [0, 1] scale prior to plotting. The baseline was determined by polynomial fitting. The DFT-computed Raman spectrum was normalized to a [0, 1] scale, and the y-axis quantity was magnified by a scale factor of 3 prior to plotting.

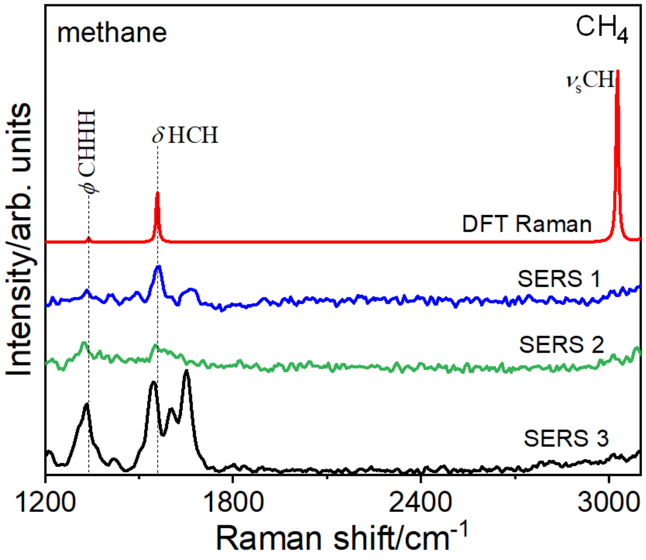

Supplementary Figure 16. Three representative examples of measured SERS spectra (blue, green, and black traces) that correspond to the detection of methane. The DFT-computed Raman vibrational spectrum, which served as the basis of the assignment of the measured SERS spectra, is also shown by the red trace. The major vibrational modes are labeled using the following symbols: ϕ-torsion; ν_s-symmetric stretching; ν_{as}-asymmetric stretching; δ-bending. Spectra are shown vertically stacked for clarity. SERS spectra were baseline-subtracted, subjected to smoothing by the Savitzsky–Golay method with a window of 5 points and a polynomial of order 3, and normalized to a [0, 1] scale prior to plotting. The baseline was determined by polynomial fitting. The DFT-computed Raman spectrum was normalized to a [0, 1] scale, and the y-axis quantity was magnified by a scale factor of 2 prior to plotting.

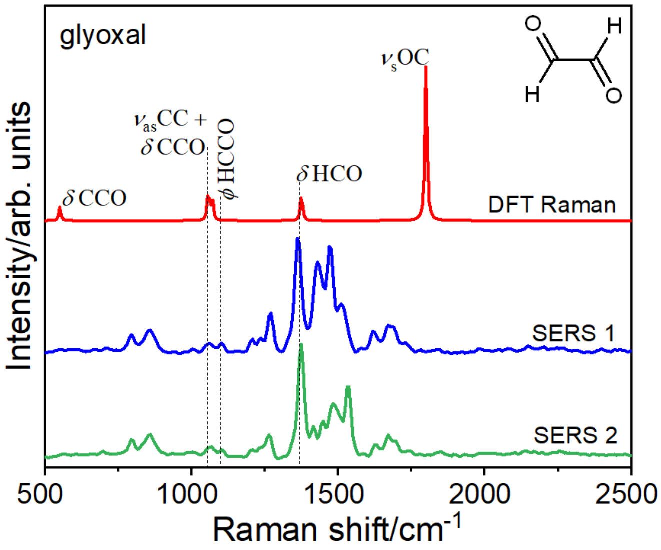

Supplementary Figure 17. Two representative examples of measured SERS spectra (blue and green traces) that correspond to the detection of glyoxal. The DFT-computed Raman vibrational spectrum, which served as the basis of the assignment of the measured SERS spectra, is also shown by the red trace. The major vibrational modes are labeled using the following symbols: ϕ-torsion; ν_s-symmetric stretching; ν_{as}-asymmetric stretching; δ-bending. Spectra are shown vertically stacked for clarity. SERS spectra were baseline-subtracted, subjected to smoothing by the Savitzky–Golay method with a window of 5 points and a polynomial of order 3, and normalized to a [0, 1] scale prior to plotting. The baseline was determined by polynomial fitting. The DFT-computed Raman spectrum was normalized to a [0, 1] scale, and the y-axis quantity was magnified by a scale factor of 3 prior to plotting.

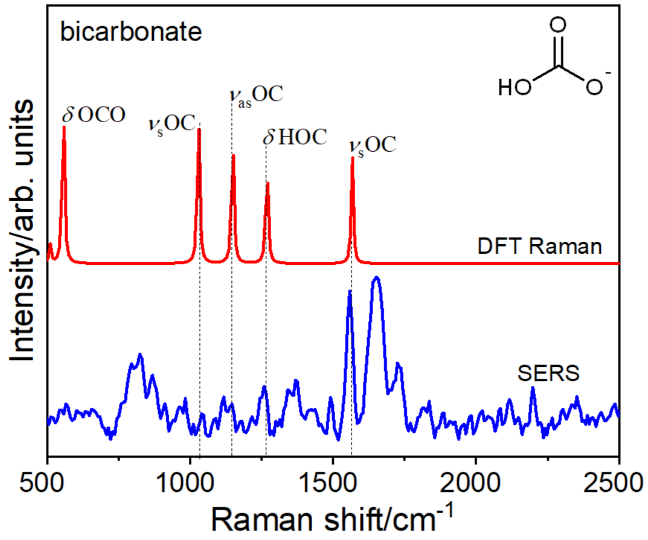

Supplementary Figure 18. One example of a measured SERS spectrum (blue trace) that corresponds to the detection of bicarbonate. The DFT-computed Raman vibrational spectrum, which served as the basis of the assignment of the measured SERS spectrum, is also shown by the red trace. The major vibrational modes are labeled using the following symbols: ϕ-torsion; ν_s-symmetric stretching; ν_{as}-asymmetric stretching; δ-bending. Spectra are shown vertically stacked for clarity. SERS spectrum was baseline-subtracted, subjected to smoothing by the Savitzsky–Golay method with a window of 5 points and a polynomial of order 3, and normalized to a [0, 1] scale and the y-axis quantity was magnified by a scale factor of 2 prior to plotting. The baseline was determined by polynomial fitting. The DFT-computed Raman spectrum was normalized to a [0, 1] scale prior to plotting.

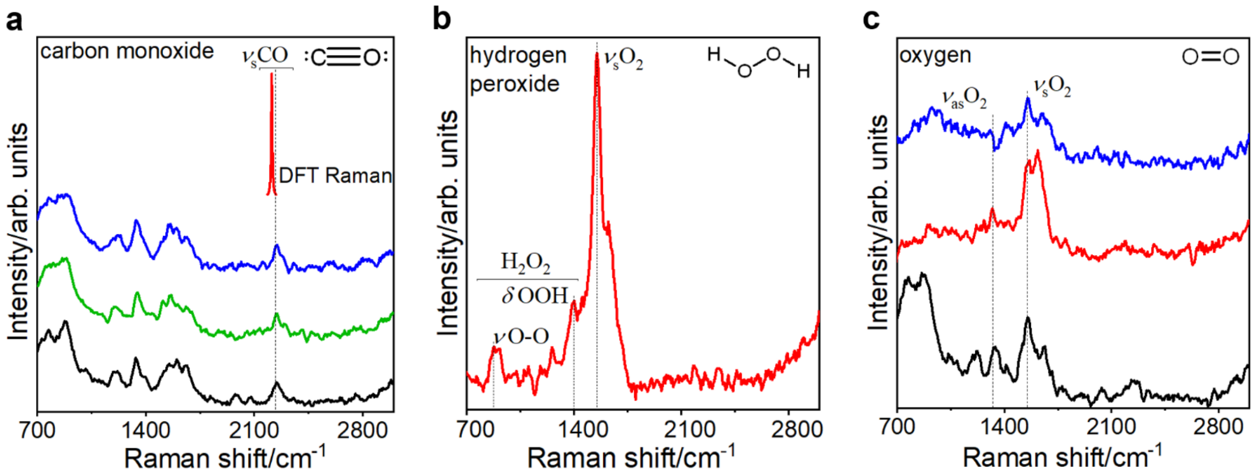

Supplementary Figure 19. a) Three representative examples of measured SERS spectra (blue, green, and black traces) that correspond to the detection of carbon monoxide. The DFT-computed Raman vibrational spectrum for carbon monoxide, which has a single mode, is also shown by the red trace. Although the ν_s mode of carbon monoxide is predicted by DFT to have a frequency of 2200 cm^{-1}, in practice, the mode can appear in the 1800–2200 cm^{-1} range depending on the adsorption motif of carbon monoxide. SERS spectra were subjected to smoothing by the Savitzsky–Golay method with a window of 5 points and a polynomial of order 1, and normalized to a [0, 1] scale and the y-axis quantity was magnified by a scale factor of 2 prior to plotting in a vertically stacked manner. The DFT-computed Raman spectrum was normalized to a [0, 1] scale prior to plotting. b) One representative example of a measured SERS spectrum (red trace) that is assigned to hydrogen peroxide.[6] The SERS spectrum was baseline-subtracted, and subjected to smoothing by the Savitzsky–Golay method with a window of 5 points and a polynomial of order 1 prior to plotting. The baseline was determined by polynomial fitting. c) Three representative examples of measured SERS spectra (blue, red, and black traces) that are assigned to oxygen.[7, 8] SERS spectra were normalized to a [0, 1] scale and subjected to smoothing by the Savitzsky–Golay method with a window of 5 points and a polynomial of order 1 prior to plotting in a vertically-stacked manner. SERS bands from vibrational modes are labeled using the following symbols: ϕ-torsion; ν-stretching; ν_s-symmetric stretching; ν_{as}-asymmetric stretching; δ-bending.

IV. Isotopological shifts and spectral barcodes

By use of ^{13}C-labeling, we attempted to confirm whether C_1–C_4 surface species detected in SERS spectra are indeed formed by CO_2RR. Species generated from $^{13}CO_2$ are expected to have vibrational spectra with their mass-sensitive modes shifted in frequency with respect to the ^{12}C isotopologs. For each species detected in experimental SERS spectra, we determined these isotopological shifts from SERS spectra measured in $^{12}CO_2$-saturated water and $^{13}CO_2$-saturated water. We compared these measured shifts with those estimated from DFT-computed Raman spectra.

Isotopological shifts: From experiments conducted in $^{12}CO_2$–saturated water, all SERS spectra corresponding to a particular species were grouped together. From each experimental SERS spectrum, the peak wavenumbers (cm^{-1}) of the key vibrational modes were determined (Supplementary Fig. 20). Note that the key vibrational modes were identified on the basis of DFT-computed Raman frequencies. Then, for each mode, we determined the average and standard deviation (SD) of the peak wavenumber, $\nu(^{12}C)$, across the entire set of experimental spectra corresponding to this species. The same procedure was followed for the set of SERS spectra corresponding to this species acquired in $^{13}CO_2$–saturated water. The latter yielded for each mode, the average and SD of the peak wavenumber, $\nu(^{13}C)$, for the heavier isotopolog. We then determined the experimental isotopological shift, $\nu(^{13}C) - \nu(^{12}C)$, and the SD in this shift for all key modes for each species. The SD in the measured isotopological shift of a mode was calculated as $\sqrt{\sigma_{12C}^2 + \sigma_{13C}^2}$, where σ_{12C} and σ_{13C} are the standard deviations in the peak wavenumbers of the mode as determined from experimental SERS spectra of the ^{12}C and ^{13}C isotopologs, respectively. These experimentally measured isotopological shifts were then compared with corresponding DFT-computed isotopological shifts for each species, as shown in Supplementary Tables 25–45.

Note that the averaging procedure described above only utilized wavenumber locations of vibrational peaks in spectra; peak intensities were disregarded because they do not provide any information on the isotopological shift.

Construction of spectral barcodes: For visualization of the isotopological shifts, measured SERS spectra of ^{12}C and ^{13}C isotopologs of each species are plotted in the form of spectral barcodes (Fig. 3 and Supplementary Figs. 21–25). To construct a spectral barcode for a species, we plotted along the x-axis unit-height vertical lines corresponding to the average peak wavenumber locations of the key vibrational modes for that species. These average peak wavenumber locations were determined from experimental SERS spectra, as described in the previous paragraph. The spectral barcodes for the ^{12}C and ^{13}C isotopologs of each species were overlaid for visualization of the experimentally measured isotopological shift. The number of experimental SERS spectra that were used to construct a barcode is listed in each case. Reference barcodes were also constructed from the DFT-computed Raman spectra of the ^{12}C and ^{13}C isotopologs of the species and overlaid for visualization of the DFT-computed isotopological shift. Supplementary Fig. 20 depicts the procedure for the construction of spectral barcodes with the aid of the example of propane.

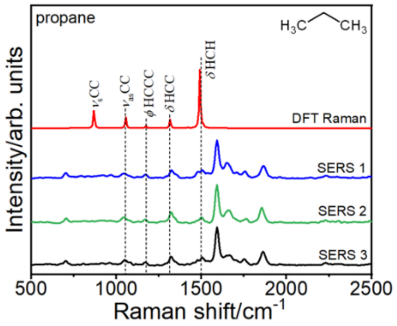

	ν CC	ϕ HCCC	δ HCC	δ HCH
**SERS 1**	1062.0	1174.8	1334.4	1511.5
**SERS 2**	1054.9	1174.8	1334.4	1511.5
**SERS 3**	1054.9	1181.8	1334.4	1504.7
⋮	⋮	⋮	⋮	⋮
**average**	1057.2	1177.1	1334.4	1509.2

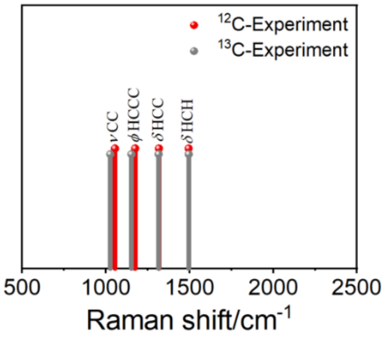

Supplementary Figure 20. Depiction of the method of construction of a spectral barcode from experimental SERS spectra. The example shown here corresponds to the ^{12}C isotopolog of propane. In the top left panel, reproduced from Supplementary Fig. 13c, three representative examples of experimental SERS spectra (blue, green, and black traces) are shown plotted along with the DFT-computed Raman vibrational spectrum (red trace), which served as the basis of the assignment of the measured SERS spectra to propane. The major vibration modes are labeled using the following symbols: ϕ-torsion; ν- stretching; δ-bending. Spectra are shown vertically stacked for clarity. SERS spectra were baseline-subtracted, subjected to smoothing by the Savitzsky–Golay method with a window of 5 points and a polynomial of order 3, and normalized to a [0, 1] scale prior to plotting. The baseline was determined by polynomial fitting. The DFT-computed Raman spectrum was normalized to a [0, 1] scale, and the y-axis quantity was magnified by a scale factor of 10 prior to plotting. From each experimental SERS spectrum corresponding to a given species, peak wavenumbers (cm^{-1}) of all key modes were determined, as shown by the table on the right. For each mode, the average of the peak wavenumber across all experimental spectra was obtained. A barcode, shown in the lower left panel, was then constructed by plotting vertical sticks corresponding to the locations of the averaged peak wave-numbers for all modes of the species. While only the process used for construction of the ^{12}C isotopolog barcode (red lines) is shown here, similar steps were employed to construct the barcode for the ^{13}C isotopolog (gray lines).

Supplementary Table 25. Experimentally measured isotopological shifts, $\nu(^{13}C) - \nu(^{12}C)$, for vibrational modes of methanol compared with corresponding DFT-computed isotopological shifts. Modes are identified by their DFT-computed frequency. The SD in the measured isotopological shift is also provided in parentheses. All values tabulated here are wavenumbers in cm^{-1} units.

mode frequency	measured shift (SD)	DFT-computed shift
1040	-16.3 (8)	-16
1072	-11.5 (7)	-8
1168	-5.4 (8)	-8
1360	-6.5 (8)	-8
1480	-12.6 (3)	-8
1496	4.2 (8)	0
1504	1.1 (7)	0

Supplementary Table 26. Experimentally measured isotopological shifts, $\nu(^{13}C) - \nu(^{12}C)$, for vibrational modes of acetic acid compared with corresponding DFT-computed isotopological shifts. Modes are identified by their DFT-computed frequency. The SD in the measured isotopological shift is also provided in parentheses. All values tabulated here are wavenumbers in cm^{-1} units.

mode frequency	measured shift (SD)	DFT-computed shift
999	-6.5 (9)	-10
1067	-24.7 (8)	-19
1205	-13.8 (7)	-15
1332	-20.9 (8)	-19
1408	-20.4 (7)	-16
1472	-3.4 (7)	-2
1818	-37.7 (4)	-45

Supplementary Table 27. Experimentally measured isotopological shifts, $\nu(^{13}C) - \nu(^{12}C)$, for vibrational modes of acetone compared with corresponding DFT-computed isotopological shifts. Modes are identified by their DFT-computed frequency. The SD in the measured isotopological shift is also provided in parentheses. All values tabulated here are wavenumbers in cm^{-1} units.

mode frequency	measured shift (SD)	DFT-computed shift
889	-16.7 (8)	-12
1083	-15.0 (8)	-12
1116	-29.4 (8)	-23
1232	-32.9 (8)	-34
1386	-13.5 (5)	-14
1461	-5.7 (5)	-2
1472	-1.4 (7)	-3
1488	-11.4 (7)	-3
1786	-40.8 (6)	-43

Supplementary Table 28. Experimentally measured isotopological shifts, $\nu(^{13}C) - \nu(^{12}C)$, for vibrational modes of butanol compared with corresponding DFT-computed isotopological shifts. Modes are identified by their DFT-computed frequency. The SD in the measured isotopological shift is also provided in parentheses. All values tabulated here are wavenumbers in cm^{-1} units.

mode frequency	measured shift (SD)	DFT-computed shift
978	-8.6 (8)	-7
1033	-27.6 (8)	-27
1072	-24.9 (7)	-24
1118	-18.7 (7)	-17
1156	-19.3 (8)	-22
1231	-11.3 (6)	-11
1257	-7.8 (8)	-7
1301	-6.2 (8)	-9
1332	-11.5 (7)	-9
1340	-8.8 (7)	-6
1403	-22.7 (8)	-19
1414	-10.7 (8)	-10
1443	-9.4 (8)	-15
1476	-9.0 (8)	-4
1496	-6.9 (8)	-2

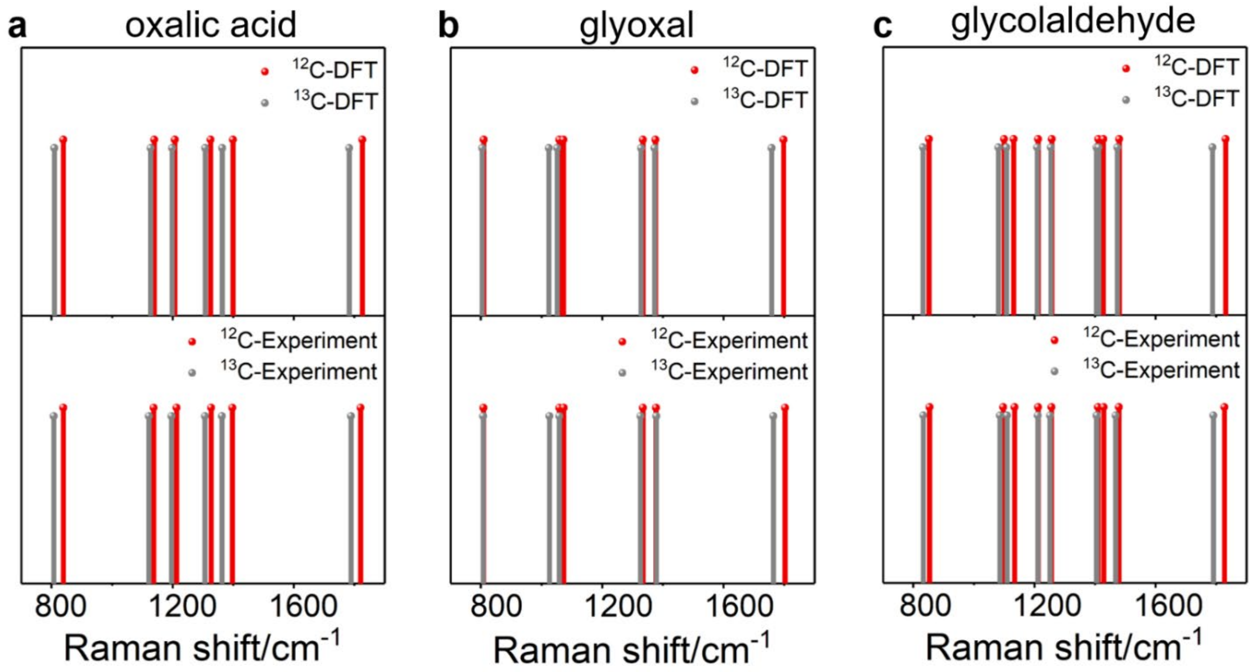

Supplementary Figure 21. Isotopological validation of CO_2RR origin of surface species. Spectral barcodes for the DFT-computed Raman spectra (top row) and experimental SERS spectra (bottom row) for ^{12}C (red lines) and ^{13}C (gray lines) isotopologs of a) oxalic acid, b) glyoxal, and c) glycolaldehyde. Each vertical line in a barcode indicates the peak wavenumber of a vibrational mode. The number of SERS spectra that led to the experimental barcodes are a) 97 and 25, b) 17 and 27, and c) 229 and 231 for the ^{12}C and ^{13}C isotopologs, respectively.

Supplementary Table 29. Experimentally measured isotopological shifts, $v(^{13}C) - v(^{12}C)$, for vibrational modes of oxalic acid compared with corresponding DFT-computed isotopological shifts. Modes are identified by their DFT-computed frequency. The SD in the measured isotopological shift is also provided in parentheses. All values tabulated here are wavenumbers in cm^{-1} units.

mode frequency	measured shift (SD)	DFT-computed shift
839	-31.7 (9)	-29
1139	-15.5 (8)	-12
1207	-16.6 (7)	-9
1325	-20.3 (8)	-17
1399	-34.9 (8)	-35
1826	-30.7 (7)	-43

Supplementary Table 30. Experimentally measured isotopological shifts, $\nu(^{13}C) - \nu(^{12}C)$, for vibrational modes of glycolaldehyde compared with corresponding DFT-computed isotopological shifts. Modes are identified by their DFT-computed frequency. The SD in the measured isotopological shift is also provided in parentheses. All values tabulated here are wavenumbers in cm^{-1} units.

mode frequency	measured shift (SD)	DFT-computed shift
852	-20.6 (8)	-20
1100	-10.5 (8)	-19
1131	-24.6 (6)	-23
1212	-1.3 (8)	-3
1257	-5.1 (8)	-3
1411	-5.5 (7)	-6
1428	-22.1 (7)	-16
1480	-10.5 (7)	-6
1831	-35.7 (7)	-43

Supplementary Table 31. Experimentally measured isotopological shifts, $\nu(^{13}C) - \nu(^{12}C)$, for vibrational modes of glyoxal compared with corresponding DFT-computed isotopological shifts. Modes are identified by their DFT-computed frequency. The SD in the measured isotopological shift is also provided in parentheses. All values tabulated here are wavenumbers in cm^{-1} units.

mode frequency	measured shift (SD)	DFT-computed shift
810.0	-1.3 (7)	-5
1059	-32.0 (6)	-35
1072	-15.1 (6)	-20
1334	-8.4 (6)	-6
1376	0.5(8)	-3
1798	-39.2 (0)	-40

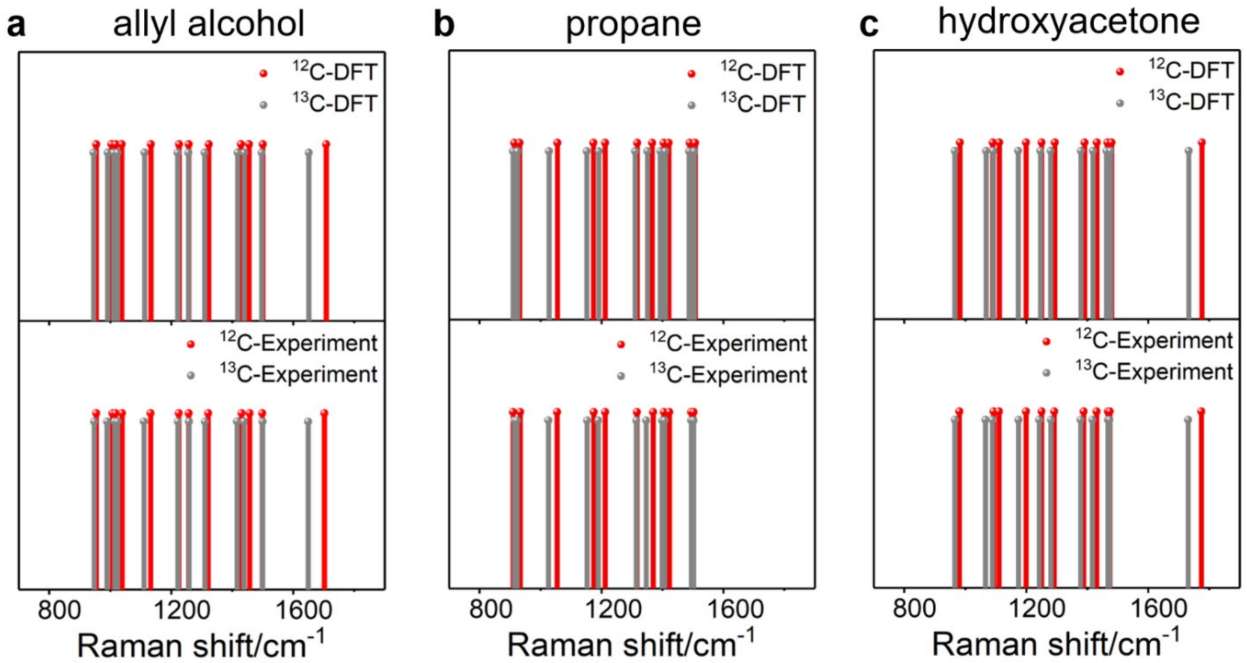

Supplementary Figure 22. Isotopological validation of CO_2RR origin of surface species. Spectral barcodes for the DFT-computed Raman spectra (top row) and experimental SERS spectra (bottom row) for ^{12}C (red lines) and ^{13}C (gray lines) isotopologs of a) allyl alcohol, b) propane, and c) hydroxyacetone. Each vertical line in a barcode indicates the peak wavenumber of a vibrational mode. The number of SERS spectra that led to the experimental barcodes are a) 352 and 516, b) 354 and 569, and c) 372 and 157 for the ^{12}C and ^{13}C isotopologs, respectively.

Supplementary Table 32. Experimentally measured isotopological shifts, $\nu(^{13}C) - \nu(^{12}C)$, for vibrational modes of allyl alcohol compared with corresponding DFT-computed isotopological shifts. Modes are identified by their DFT-computed frequency. The SD in the measured isotopological shift is also provided in parentheses. All values tabulated here are wavenumbers in cm^{-1} units.

mode frequency	measured shift (SD)	DFT-computed shift
954	-6.2 (8)	-9
1004	-18.2 (8)	-12
1018	-2.6 (8)	-7
1038	-15.3 (8)	-14
1133	-21.6 (8)	-20
1226	-2.9 (8)	-5
1257	1.6 (9)	-1
1323	-11.2 (6)	-13
1429	-13.9 (8)	-11
1455	-18.4 (8)	-15
1501	1.6 (8)	-4
1708	-52.8 (7)	-57

Supplementary Table 33. Experimentally measured isotopological shifts, $\nu(^{13}C) - \nu(^{12}C)$, for vibrational modes of propane compared with corresponding DFT-computed isotopological shifts. Modes are identified by their DFT-computed frequency. The SD in the measured isotopological shift is also provided in parentheses. All values tabulated here are wavenumbers in cm^{-1} units.

mode frequency	measured shift (SD)	DFT-computed shift
914	4.8 (8)	-4
932	-11.3 (8)	-7
1056	-28.1 (8)	-28
1174	-22.2 (8)	-22
1212	-21.8 (8)	-21
1317	-2.0 (6)	-6
1367	-22.8 (8)	-16
1404	-5.8 (7)	-11
1421	-15.8 (8)	-11
1490	0.6 (7)	-2
1507	0.6 (8)	-2

Supplementary Table 34. Experimentally measured isotopological shifts, $v(^{13}C) - v(^{12}C)$, for vibrational modes of hydroxyacetone compared with corresponding DFT-computed isotopological shifts. Modes are identified by their DFT-computed frequency. The SD in the measured isotopological shift is also provided in parentheses. All values tabulated here are wavenumbers in cm^{-1} units.

mode frequency	measured shift (SD)	DFT-computed shift
983	-15.7 (7)	-17
1090	-25.9 (8)	-22
1112	-21.6 (8)	-20
1199	-24.3 (6)	-25
1251	-8.3 (8)	-4
1294	-10.7 (8)	-14
1391	-10.1 (7)	-12
1432	-13.3 (9)	-13
1466	-0.7 (7)	-3
1480	-0.9 (7)	-3
1776	-42.9 (8)	-43

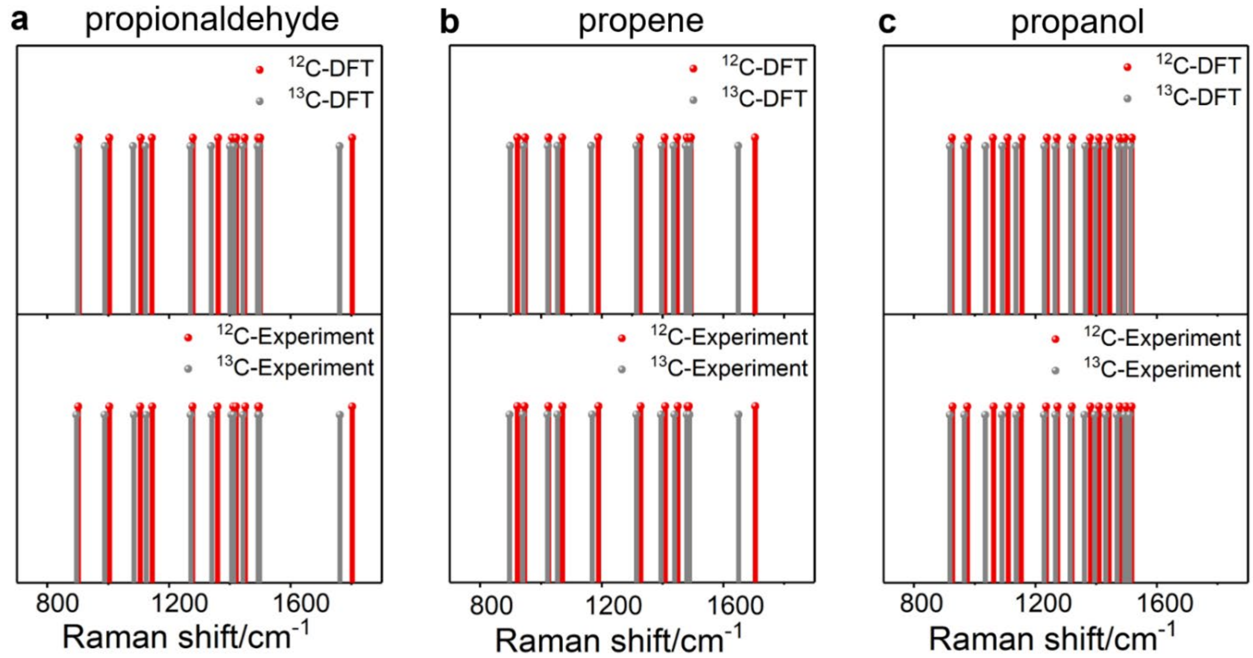

Supplementary Figure 23. Isotopological validation of CO_2RR origin of surface species. Spectral barcodes for the DFT-computed Raman spectra (top row) and experimental SERS spectra (bottom row) for ^{12}C (red lines) and ^{13}C (gray lines) isotopologs of a) propionaldehyde, b) propene, and c) propanol. Each vertical line in a barcode indicates the peak wavenumber of a vibrational mode. The number of SERS spectra that led to the experimental barcodes are a) 598 and 312, b) 316 and 513, and c) 666 and 684 for the ^{12}C and ^{13}C isotopologs, respectively.

Supplementary Table 35. Experimentally measured isotopological shifts, $\nu(^{13}C) - \nu(^{12}C)$, for vibrational modes of propionaldehyde compared with corresponding DFT-computed isotopological shifts. Modes are identified by their DFT-computed frequency. The SD in the measured isotopological shift is also provided in parentheses. All values tabulated here are wavenumbers in cm^{-1} units.

mode frequency	measured shift (SD)	DFT-computed shift
905	-5.1 (8)	-5
1004	-15.4 (8)	-14
1108	-20.7 (6)	-26
1144	-19.2 (8)	-21
1279	-5.6 (8)	-8
1362	-18.4 (8)	-23
1408	-6.3 (8)	-7
1422	-7.6 (8)	-7
1449	-6.9 (8)	-6
1494	0.6 (7)	-2
1501	1.1 (7)	-3
1803	-39.6 (6)	-42

Supplementary Table 36. Experimentally measured isotopological shifts, $\nu(^{13}C) - \nu(^{12}C)$, for vibrational modes of propene compared with corresponding DFT-computed isotopological shifts. Modes are identified by their DFT-computed frequency. The SD in the measured isotopological shift is also provided in parentheses. All values tabulated here are wavenumbers in cm^{-1} units.

mode frequency	measured shift (SD)	DFT-computed shift
923	-26.0 (7)	-24
948	-5.6 (8)	-5
1025	-2.6 (7)	-2
1070	-16.9 (8)	-15
1189	-19.9 (8)	-23
1326	-14.0 (8)	-11
1407	-13.8 (8)	-10
1448	-10.8 (8)	-11
1480	-1.7 (8)	-3
1494	1.9 (8)	-3
1704	-55.5 (8)	-55

Supplementary Table 37. Experimentally measured isotopological shifts, $\nu(^{13}C) - \nu(^{12}C)$, for vibrational modes of propanol compared with corresponding DFT-computed isotopological shifts. Modes are identified by their DFT-computed frequency. The SD in the measured isotopological shift is also provided in parentheses. All values tabulated here are wavenumbers in cm^{-1} units.

mode frequency	measured shift (SD)	DFT-computed shift
926	-8.3 (8)	-7
979	-10.5 (6)	-13
1061	-27.5 (9)	-25
1109	-20.3 (8)	-18
1156	-16.4 (7)	-20
1239	-6.8 (8)	-9
1271	-7.5 (7)	-6
1322	-6.6 (8)	-6
1380	-19.7 (8)	-15
1410	-14.6 (8)	-12
1444	-10.1 (8)	-15
1478	-9.3 (8)	-4
1495	-4.0 (8)	-2
1518	-7.1 (8)	-4

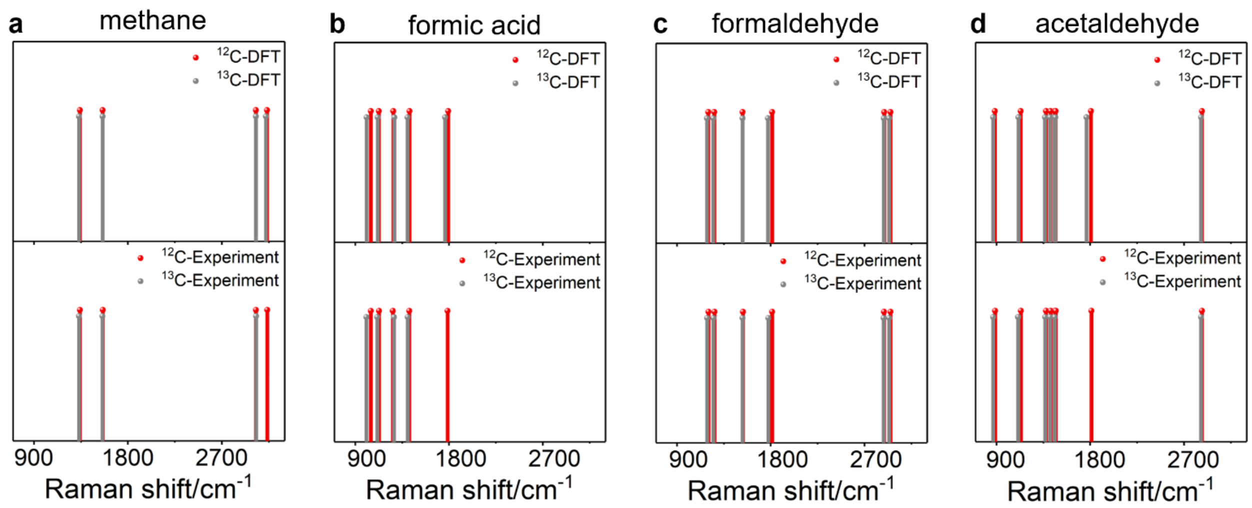

Supplementary Figure 24. Isotopological validation of CO_2RR origin of surface species. Spectral barcodes for the DFT-computed Raman spectra (top row) and experimental SERS spectra (bottom row) for ^{12}C (red lines) and ^{13}C (gray lines) isotopologs of a) methane, b) formic acid, c) formaldehyde and d) acetaldehyde. Each vertical line in a barcode indicates the peak wavenumber of a vibrational mode. The number of SERS spectra that led to the experimental barcodes are a) 6 and 3, b) 10 and 3, c) 28 and 15, and d) 100 and 316 for the ^{12}C and ^{13}C isotopologs, respectively.

Supplementary Table 38. Experimentally measured isotopological shifts, $\nu(^{13}C) - \nu(^{12}C)$, for vibrational modes of methane compared with corresponding DFT-computed isotopological shifts. Modes are identified by their DFT-computed frequency. The SD in the measured isotopological shift is also provided in parentheses. All values tabulated here are wavenumbers in cm $^{-1}$ units.

mode frequency	measured shift (SD)	DFT-computed shift
1340	-8.0 (7)	-9
1558	-3.2 (9)	0
3026	0.8 (6)	0
3132	-	-11

Supplementary Table 39. Experimentally measured isotopological shifts, $v(^{13}C) - v(^{12}C)$, for vibrational modes of formic acid compared with corresponding DFT-computed isotopological shifts. Modes are identified by their DFT-computed frequency. The SD in the measured isotopological shift is also provided in parentheses. All values tabulated here are wavenumbers in cm^{-1} units.

mode frequency	measured shift (SD)	DFT-computed shift
1050	-43.9 (5)	-41
1128	-18.3 (3)	-12
1266	9.9 (6)	10
1421	-14.1 (8)	-19
1792	-	-29

Supplementary Table 40. Experimentally measured isotopological shifts, $v(^{13}C) - v(^{12}C)$, for vibrational modes of formaldehyde compared with corresponding DFT-computed isotopological shifts. Modes are identified by their DFT-computed frequency. The SD in the measured isotopological shift is also provided in parentheses. All values tabulated here are wavenumbers in cm^{-1} units.

mode frequency	measured shift (SD)	DFT-computed shift
1201	-12.7 (8)	-12
1259	-9.2 (6)	-10
1530	-2.4 (7)	0
1813	-37.8 (8)	-40
2889	-3.6 (5)	-4
2948	-12.5 (7)	-13

Supplementary Table 41. Experimentally measured isotopological shifts, $v(^{13}C) - v(^{12}C)$, for vibrational modes of acetaldehyde compared with corresponding DFT-computed isotopological shifts. Modes are identified by their DFT-computed frequency. The SD in the measured isotopological shift is also provided in parentheses. All values tabulated here are wavenumbers in cm^{-1} units.

mode frequency	measured shift (SD)	DFT-computed shift
886	-21.0 (6)	-18
1133	-27.5 (7)	-20
1377	-8.4 (8)	-11
1420	-5.6 (6)	-5
1460	-2.9 (5)	-2
1469	-9.0 (7)	-2
1808	0.0 (0)	-43
2871	-9.4 (7)	-8

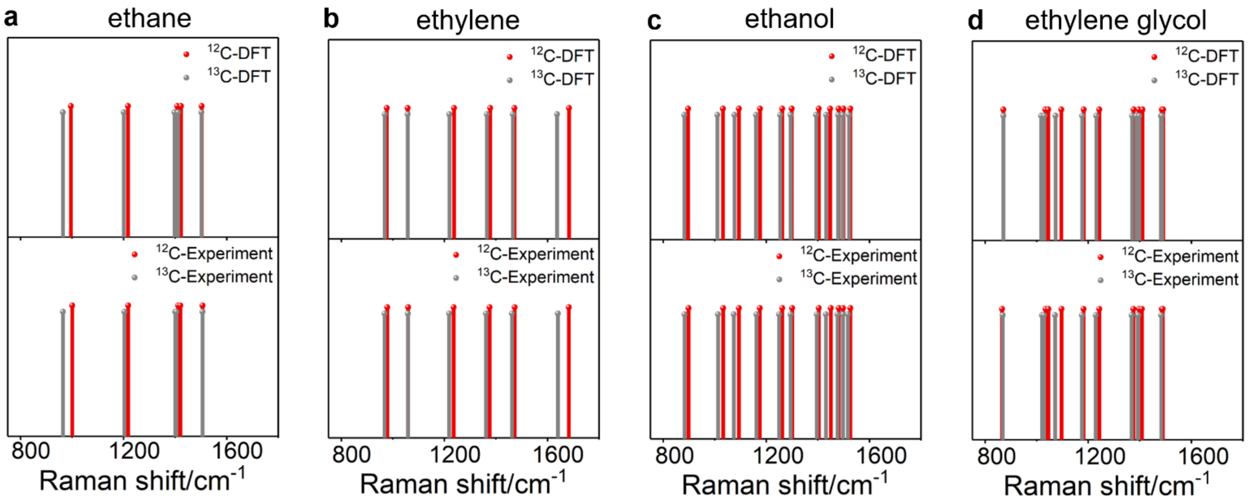

Supplementary Figure 25. Isotopological validation of CO_2RR origin of surface species. Spectral barcodes for the DFT-computed Raman spectra (top row) and experimental SERS spectra (bottom row) for ^{12}C (red lines) and ^{13}C (gray lines) isotopologs of a) ethane, b) ethylene, c) ethanol and d) ethylene glycol. Each vertical line in a barcode indicates the peak wavenumber of a vibrational mode. The number of SERS spectra that led to the experimental barcodes are a) 341 and 81, b) 49 and 146, c) 177 and 162, and d) 873 and 344 for the ^{12}C and ^{13}C isotopologs, respectively.

Supplementary Table 42. Experimentally measured isotopological shifts, $\nu(^{13}C) - \nu(^{12}C)$, for vibrational modes of ethane compared with corresponding DFT-computed isotopological shifts. Modes are identified by their DFT-computed frequency. The SD in the measured isotopological shift is also provided in parentheses. All values tabulated here are wavenumbers in cm^{-1} units.

mode frequency	measured shift (SD)	DFT-computed shift
996	-36.0 (6)	-31
1217	-14.3 (7)	-16
1408	-11.3 (7)	-10
1423	-15.8 (7)	-11
1503	0.1 (7)	-1

Supplementary Table 43. Experimentally measured isotopological shifts, $v(^{13}C) - v(^{12}C)$, for vibrational modes of ethylene glycol compared with corresponding DFT-computed isotopological shifts. Modes are identified by their DFT-computed frequency. The SD in the measured isotopological shift is also provided in parentheses. All values tabulated here are wavenumbers in cm^{-1} units.

mode frequency	measured shift (SD)	DFT-computed shift
871	3.1 (7)	0
1034	-13.4 (8)	-17
1045	-17.2 (7)	-15
1096	-25.3 (7)	-23
1183	-5.8 (8)	-6
1243	-14.2 (8)	-11
1376	-7.2 (8)	-6
1396	-6.1 (7)	-9
1411	-13.9 (7)	-13
1486	-2.6 (7)	-4
1491	-5.9 (7)	-4

Supplementary Table 44. Experimentally measured isotopological shifts, $v(^{13}C) - v(^{12}C)$, for vibrational modes of ethylene compared with corresponding DFT-computed isotopological shifts. Modes are identified by their DFT-computed frequency. The SD in the measured isotopological shift is also provided in parentheses. All values tabulated here are wavenumbers in cm^{-1} units.

mode frequency	measured shift (SD)	DFT-computed shift
977	-10.3 (8)	-8
1058	-1.2 (5)	0
1238	-16.6 (6)	-18
1378	-13.5 (8)	-13
1472	-10.5 (7)	-6
1684	-41.9 (7)	-47

Supplementary Table 45. Experimentally measured isotopological shifts, $\nu(^{13}C) - \nu(^{12}C)$, for vibrational modes of ethanol compared with corresponding DFT-computed isotopological shifts. Modes are identified by their DFT-computed frequency. The SD in the measured isotopological shift is also provided in parentheses. All values tabulated here are wavenumbers in cm^{-1} units.

mode frequency	measured shift (SD)	DFT-computed shift
897	-15.8 (9)	-15
1032	-19.0 (8)	-22
1094	-19.1 (7)	-17
1176	-12.4 (8)	-16
1262	-11.7 (6)	-8
1299	-6.1 (8)	-4
1404	-7.6 (7)	-10
1448	-18.8 (8)	-16
1481	-5.4 (7)	-2
1500	-0.1 (8)	-3
1526	-8.8 (8)	-5

V. Distribution of surface species formed in $^{13}CO_2$-saturated water

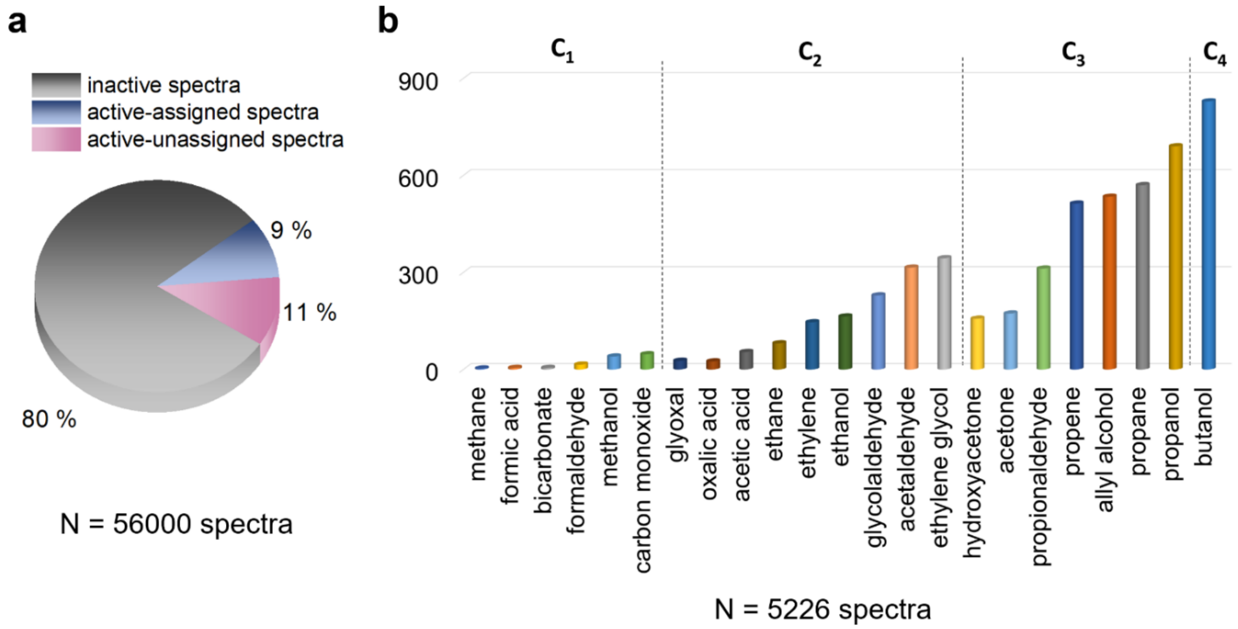

Supplementary Figure 26. a) Pie-chart showing the % distribution of inactive SERS spectra, active spectra that were assigned to a species in the database, and active spectra that were unassignable. This distribution was obtained by an analysis of 56,000 in-situ SERS spectra collected from 56 individual Ag NP scatterers in $^{13}CO_2$-saturated water under plasmonic excitation. d) Bar plot showing for each surface species the number of detection events across the set of active, assigned spectra. The species are grouped into C_1, C_2, C_3, and C_4 categories; within each category, they are listed in the order of their prevalence. N in panels a and b represents the total number of spectra that led to the plot.

VI. Analysis of photon flux

Under the continuous-wave (CW) laser excitation conditions employed in our SERS experiments, we estimated the average time between individual photon absorption events by a Ag NP scatterer. We assumed the scatterer to be a dimer of 60-nm diameter spherical Ag NPs, which has an estimated absorption cross-section, σ, of 5.12 x 10^{-15} m^2 at 514.5 nm.[9]

Supplementary Table 46. Parameters and expressions used for the determination of the average time between photon absorption events.

Parameters	Equations	Magnitude	Units
excitation wavelength (λ)		514.5	nm
photon energy	$E = \frac{hc}{\lambda}$	2.41	eV
photon energy (E)	1 eV = 1.6 x 10^{-19} J	3.86 x 10^{-19}	J
laser power (P)		10	mW
beam diameter (φ)		10.8	μm
beam area (A)	$A = \pi \left(\frac{\varphi}{2}\right)^2$	9.15 x 10^{-11}	m^2
intensity (I)	$I = P/A$	1.09 x 10^{8}	$W.m^{-2}$
absorption cross-section (σ)		5.12 x 10^{-15}	m^2
absorbed power (P_{abs})	$P_{abs} = I.\sigma$	5.58 x 10^{-7}	W
rate of photon absorption (R_{abs})	$R_{abs} = P_{abs}/E$	1.45 x 10^{12}	s^{-1}
average time between photon absorption events (τ)	$\tau = 1/R_{abs}$	6.90 x 10^{-13}	s
		690	fs

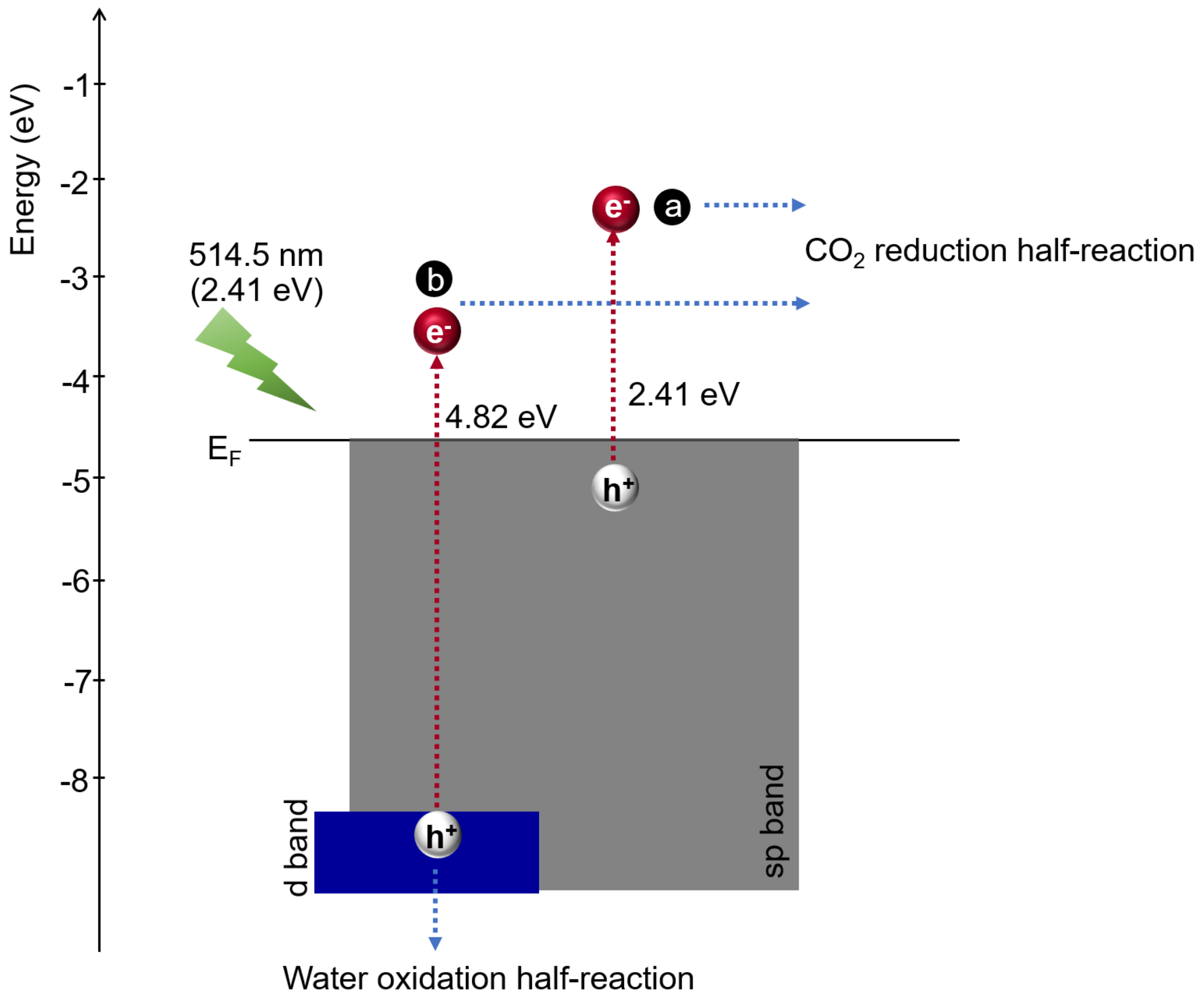

Supplementary Figure 27. Energy diagram depicting possible carrier excitations in a Ag NP dimer, initiated by 514.5 nm laser excitation (2.41 eV). In a typical scenario (depicted as "a"), an LSPR excited in a Ag NP by visible light would undergo decay via an intraband transition to form an electron–hole pair in the sp band of Ag. The resulting electron is considerably energetic relative to the Fermi level (E_F) of Ag, but the hole is not sufficiently energetic for water oxidation, which has a standard reduction potential of 1.23 V vs the standard hydrogen electrode at a pH of 0, i.e., –5.67 V relative to vacuum.[10] Interband transitions cannot be excited in Ag by 514.5 nm laser excitation. Due to the ~4 eV interband edge of Ag,[11] UV light excitation is required. However, the focused laser excitation conditions we employ (Supplementary Table 46) can favor multiphoton excitation and carrier re-excitation processes, which would result in the generation of highly energetic electron–hole pairs akin to those generated by UV light. For instance, two-photon absorption or energetic combination of two sp-band electron–hole pairs of 2.41 eV energy each can induce an interband transition (depicted as "b") generating a hole in the d band. Such a hole would be energetic enough for the water oxidation half-reaction. Energies are shown relative to the vacuum level.

VII. Calculation of temperature rise resulting from photothermal heating

Under CW laser excitation, a Ag NP scatterer will undergo photothermal heating. As a result of this heating, the NP surface is expected to be at steady-state temperature that is higher than the bulk temperature of the ambient medium by an amount ΔT. We estimated ΔT under our experimental conditions: CW excitation with a 514.5 nm laser with a power of 10 mW and a beam diameter of 10.8 μm. The focused laser spot excites only a discrete scatterer at a time, since scatterers on the substrate are well separated from one another (Supplementary Figs. 4 and 5). Therefore, we use an expression obtained from the steady-state heat-balance analysis of a single absorbing entity:[9, 12]

$$\Delta T = \frac{\sigma I}{4\pi r \kappa} \qquad (1)$$

In this equation, I is the laser intensity estimated to be 1.09 x 10^8 W.m^{-2} (Supplementary Table 46). σ is the absorption cross-section of the absorbing entity, which we assume to be a dimer of 60-nm diameter Ag NPs[9] for a reasonable representation of a typical scatterer interrogated in our studies (Supplementary Figs. 4–7). σ for a dimer of 60-nm diameter Ag NPs has an estimated value of 5.12 x 10^{-15} m^2 at the excitation wavelength of 514.5 nm.[9] r is the radius of the absorber, which is effectively 37.8 nm based on the total volume of the dimer. κ is the thermal conductivity of the surrounding medium, which has a value of 0.6 W.m^{-1}.K^{-1} for water, which overlies the NP dimer, and a value of 1.2 W m^{-1} K^{-1} for the borosilicate glass coverslip that underlies the NP dimer. For the worst-case scenario of photothermal heating, we use the lower value of κ. Substituting these values into equation 1, ΔT is determined to be 2 K. In other words, the steady-state local temperature at the surface of the NP scatterer is 2 K higher than the bulk temperature (296 K) of the aqueous medium. Alternatively, we make an estimate using an effective thermal conductivity,

κ_{eff}, that is a simple average of the κ values of glass and water. In such a case, κ_{eff} is 0.9 W. m^{-1}. K^{-1} and the estimated temperature rise is 1.3 K. Such a small photoinduced temperature rise (2K or smaller) cannot be assigned responsibility for the observed CO_2RR activity.[13]

VIII. Supplementary Movie Captions:

Supplementary Movie 1: Movie of in situ SERS spectra acquired in situ from an individual Ag NP scatterer in water that is not intentionally saturated with CO_2 under focused 514.5 nm laser excitation. SERS spectra were acquired continuously with a 60× microscope objective with an acquisition time of 200 ms per frame. No significant vibrational features or dynamics were observed in the spectra in this control experiment.

Supplementary Movie 2. Movie of in situ SERS spectra acquired from an individual Ag NP scatterer in $^{12}CO_2$-saturated water under focused 514.5 nm laser excitation. SERS spectra were acquired continuously with a 60× microscope objective with an acquisition time of 200 ms per frame. The spectra acquired under these photocatalytic CO_2RR conditions showed distinct vibrational bands that appear and disappear from one frame to another. This dynamics indicates the formation of transient species at the surface of the Ag.

IX. Supplementary References

1. Lee, P. C. & Meisel, D. Adsorption and surface-enhanced Raman of dyes on silver and gold sols. *J. Phys. Chem.* **86**, 3391–3395 (1982).
2. N. Waterhouse, G. I., A. Bowmaker, G. & B. Metson, J. The thermal decomposition of silver (I, III) oxide: a combined XRD, FT-IR and Raman spectroscopic study. *Phys. Chem. Chem. Phys.* **3**, 3838–3845 (2001).
3. Martina, I. Micro-Raman characterisation of silver corrosion products: Instrumental set up and reference database. 8 (2012).
4. Jamróz, M. H. Vibrational Energy Distribution Analysis (VEDA): scopes and limitations. *Spectrochim. Acta. A. Mol. Biomol. Spectrosc.* **114**, 220–230 (2013).
5. Michal. H. Jamroz. *Vibrational Energy Distribution Analysis VEDA 4*. (Warsaw, 2004-2010).
6. Giguére, P. A. & Srinivasan, T. K. K. A Raman study of H_2O_2 and D_2O_2 vapor. *J. Raman Spectrosc.* **2**, 125–132 (1974).
7. Ricks, A. M., Douberly, G. E. & Duncan, M. A. IR photodissociation spectroscopy of O_{4+}, O_{6+} and O_{8+} cluster ions. *Int. J. Mass Spectrom.* **283**, 69–76 (2009).
8. Green, I. X. & Yates, J. T. Vibrational spectroscopic observation of weakly bound adsorbed molecular oxygen on powdered titanium dioxide. *J. Phys. Chem. C* **114**, 11924–11930 (2010).
9. Kumari, G., Zhang, X., Devasia, D., Heo, J. & Jain, P. K. Watching visible light-driven CO_2 reduction on a plasmonic nanoparticle catalyst. *ACS Nano* **12**, 8330–8340 (2018).
10. Yu, S. & Jain, P. K. Plasmonic photosynthesis of C_1–C_3 hydrocarbons from carbon dioxide assisted by an ionic liquid. *Nat. Commun.* **10**, 1–7 (2019).
11. Christensen, N. E. The band structure of silver and optical interband transitions. *Phys. Status Solidi B* **54**, 551–563 (1972).
12. Dijk, M. A. van & Natuurwetenschappen, F. der W. en. Nonlinear optical studies of single gold nanoparticles. (Leiden University, 2007).
13. Simakov, D. S. A. Thermocatalytic conversion of CO_2. in *Renewable Synthetic Fuels and Chemicals from Carbon Dioxide: Fundamentals, Catalysis, Design Considerations and Technological Challenges* (ed. Simakov, D. S. A.) 1–25 (Springer International Publishing, 2017).
