# Supplementary material for: A rich catalog of C–C bonded species formed in CO2 reduction on a plasmonic photocatalyst
Source: Nat Commun. 2021 May 10;12:2612. doi: 10.1038/s41467-021-22868-9 (PMC8110802; doi:10.1038/s41467-021-22868-9)
Supplement: Supplementary file 4 — Description of Additional Supplementary Files [file 41467_2021_22868_MOESM4_ESM.docx]

Description of additional supplementary information

Title: Supplementary Movie 1

Description: Movie of in situ SERS spectra acquired from an individual Ag NP scatterer in water that is not intentionally saturated with CO_2_ under focused 514.5 nm laser excitation. SERS spectra were acquired continuously with a 60× microscope objective with an acquisition time of 200 ms per frame. No significant vibrational features or dynamics were observed in the spectra in this control experiment.

Title: Supplementary Movie 2.

Description: Movie of in situ SERS spectra acquired from an individual Ag NP scatterer in ^12^CO_2_-saturated water under focused 514.5 nm laser excitation. SERS spectra were acquired continuously with a 60× microscope objective with an acquisition time of 200 ms per frame. The spectra acquired under these photocatalytic CO_2_RR conditions showed distinct vibrational bands that appear and disappear from one frame to another. This dynamics indicates the formation of transient species at the surface of the Ag.
